# Lethal interactions among forest‐grouse predators are numerous, motivated by hunger and carcasses, and their impacts determined by the demographic value of the victims

**DOI:** 10.1002/ece3.7574

**Published:** 2021-05-02

**Authors:** Cristian N. Waggershauser, Lise Ruffino, Kenny Kortland, Xavier Lambin

**Affiliations:** ^1^ School of Biological Sciences University of Aberdeen Aberdeen UK; ^2^ Joint Nature Conservation Committee Aberdeen UK; ^3^ Forestry and Land Scotland Inverness UK

**Keywords:** conservation, forest‐grouse, intraguild predation, killing, predator interactions, suppression

## Abstract

New vertebrate communities are emerging in Europe following the recovery of multiple native predators to highly anthropized landscapes where predator control is still prevalent. While the lack of reference points for these communities creates novel challenges for conservationists and wildlife managers, they also provide opportunities to further our understanding of species interactions. Despite a growing body of evidence, many aspects of interactions among predators remain poorly understood, impairing our ability to anticipate the effects of such changes in predator communities. Through a systematic literature review, we gathered all the available evidence concerning the existence, strength, and demographic impacts of lethal predator interactions among forest‐grouse predators in Europe. We found a highly interconnected predator community, with 44 pairwise lethal interactions among 12 taxa. Three of these resulted in some degree of population suppression of the victim, while another three did not. However, most interactions (38) have not been evaluated for population suppression. Additionally, we highlight how predators interact simultaneously with a large range of other predators and identified at least two further taxa possibly suppressed through the combined impacts of multiple predators. We propose that interactions causing demographic suppression are characterized by impacts on individuals with high survival elasticity and that they are motivated by food limitation and additionally, in mammals, by competition for carcasses. Predator interactions, and our still poor understanding of them, introduce large uncertainties to conservation actions based on the management of predator abundances, which should be carefully evaluated.

## Graphical abstract

The uncertainties surrounding emerging predator communities in Europe and the resulting networks of predator interactions create novel challenges for conservation and wildlife management but also offer new opportunities to improve our understanding of species ecology and interactions. Through a literature review of the interactions among the predators of forest grouse in Europe, we found a highly interconnected community of predators, with at least 44 interactions among 12 taxa, three of which result in some degree of population suppression. While the frequency of predator interactions is seemingly driven by food limitation and, in mammals, by competition for carcasses, we propose that whether they cause suppression or not is determined by whether impacts are on population classes whose survival has large effects on the population growth rate.

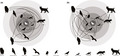

## 1 INTRODUCTION

Contemporary and historical legal and illegal persecution of vertebrate predators as well as declines of their prey's abundance, changes in land‐use, pollution, and the impact of persistent chemicals in food chains have historically reduced their populations in Europe (Breitenmoser, 1998; Fernández & deAzua, 2010; Helander et al., 2009; Newton, 1979; Ratcliffe, 1970). More recently, despite the fact that many remain largely absent throughout the European continent and threats persist across their range, some of these avian and mammalian predators are expanding in range or abundance (e.g., Eurasian lynx *Lynx lynx*, golden eagles *Aquila chrysaetos*; Chapron et al., 2014; Deinet et al., 2013; Eaton et al., 2007; Sainsbury et al., 2019). Legal protection, increasing abundances of ungulate species, and reintroductions combined with rural depopulation, farm abandonment as well as declines in the censuses of active hunters have arguably contributed to the recovery of these native predators in Europe (Linnell et al., 2009; Linnell & Zachos, 2011; Massei et al., 2014; Navarro & Pereira, 2012; Trouwborst, 2010).

Concomitantly, other European predators such as red foxes (*Vulpes vulpes*) and carrion crows (*Corvus corone*) remain the target of sustained population control efforts (Bolton et al., 2007; Kämmerle & Storch, 2019). Lethal control of predators to achieve desired ecosystem states is a prevailing management practice in Europe and beyond (Allen & Fleming, 2004; Breitenmoser, 1998; Reynolds & Tapper, 1996; Saunders & Harris, 1993). Historically aimed at the protection of livestock and game species, more recently, lethal control of predators has also been implemented for the protection of threatened prey species (Reynolds & Tapper, 1996). The return of some predators to some former areas of their range combined with the attempts to control others, showcases the existence of understudied ecological communities within highly anthropized landscapes. While predicting the outcomes of animal interactions in changing communities lacking clear ecological baselines is a daunting task (Pires, 2017; Ritchie et al., 2012), these communities offer exciting opportunities to further our understanding of species ecology and interactions (e.g., Cunningham et al., 2020; Lindström et al., 1994).

Competitive and predator–prey interactions have received considerable attention in the scientific literature (Barbosa & Castellanos, 2005; Fedriani et al., 2000; Gorini et al., 2012; Holling, 1959; Putman, 1994; Wiens, 1993). In this study, we focus on interactions between predators, specifically those of lethal nature. There is a growing body of evidence highlighting the widespread prevalence of lethal interactions between predators such as interspecific killing (killing another predator without consuming it) and intraguild predation (killing and consuming another predator of the same guild), hereafter collectively referred to as predator interactions (Lourenço et al., 2011; Palomares & Caro, 1999; Prugh & Sivy, 2020). Predator interactions are ubiquitous between both mammalian and avian predators, and they can account for large portions of the victim species’ mortality and suppress their abundance or modify their behavior and habitat use (Brashares et al., 2010; Linnell & Strand, 2000; Prugh & Sivy, 2020; Prugh et al., 2009; Sergio & Hiraldo, 2008). The evidence of their wider ecological implications in vertebrate terrestrial systems, however, remains mixed, and biased toward North American case studies involving coyotes (*Canis latrans*), as either top or mesopredator (Brashares et al., 2010; Jachowski et al., 2020). In some instances, such as the alleged protection that dingoes (*Canis lupus dingo*) confer upon Australian marsupials through the suppression of invasive mesopredators, the evidence is highly contended (Allen et al., 2011, 2015; Fancourt et al., 2019). Therefore, it is largely uncertain how widely applicable such effects are. Indeed, the strength and outcome of predator interactions are contingent on factors such as food availability (Lourenço et al., 2018), ecosystem productivity (Elmhagen et al., 2010; Elmhagen & Rushton, 2007), or the complexity of predator communities (Finke & Denno, 2004; Prugh et al., 2009). We took the opportunity to appraise a large proportion of the evidence of predator interactions in Europe and present a synthetic view of their occurrence and of the ecological processes involved.

This review was centered around the predators of forest grouse (in Europe: capercaillie *Tetrao urogallus*, hazel grouse *Tetrastes bonasia*, black grouse *Lyrurus tetrix*, and willow grouse *Lagopus lagopus*). Forest grouse and their predators are a widely studied group across Europe, and as such are a high‐profile case study relevant to both contemporary conservation and game and predator management issues. Traditionally, forest grouse were considered game species and also pests in forestry operations (Moss et al., 1979; Palmer, 1965; Stevenson, 2007). However, they are now the target of conservation efforts owing to severe declines of abundance and range contractions (Storch, 2007). The drivers of these declines are many and include habitat loss and fragmentation, climate change, as well as elevated predation by abundant generalist mesopredators (Kämmerle et al., 2017; Kurki et al., 1997; Moss et al., 2001; Selås et al., 2011). In response to the latter, predator control is often prescribed (Kämmerle & Storch, 2019; Summers et al., 2004). Concurrently, various potential grouse predators such as northern goshawks (*Accipiter gentilis*), eagle owls (*Bubo bubo*), pine martens (*Martes martes*), or Eurasian lynx have undergone local or regional expansions of their ranges in Europe (Chapron et al., 2014; Hoy et al., 2017; Mueller et al., 2016; Sheehy et al., 2018). Forest grouse and their predators are, thus, a well‐suited case study to explore the role predator interactions play in changing ecological communities.

In this study, we summarize and evaluate scattered evidence of predator interactions among forest‐grouse predators through a review of the literature. Specifically, we assess the evidence of (i) grouse predators killing or eating intraguild predators; (ii) the proportion of intraguild prey killed; and (iii) population impacts of predator interactions on intraguild prey. We weigh the strength of the evidence available for such interactions, identifying those likely causing demographic impacts on victim species from those unlikely to, and those for which we lack data. We also assess the possible drivers of such interactions and their outcomes, putting forward a hypothesis to discern between predator interactions with potential to cause suppression of the victim population from those without it. We identify knowledge gaps to improve our understanding of predator interactions and conclude with a short remark regarding the implications of our current level of understanding of predator interactions for the conservation of forest grouse and vulnerable prey species.

## 2 METHODS

So‐called European forest‐grouse species are not strictly restricted to forest habitats. Their reliance on such habitats varies throughout their range, as does the community of predators they are exposed to. Consequently, we have loosely circumscribed our review to boreal and temperate regions of Europe, limited by the tundra in the north, the Pyrenees, Alps, and Carpathian Mountains in the south and the Ural Mountains to the east. Doing so, we encompass the remaining populations of forest grouse in Europe while maintaining a relatively stable community of grouse predators.

In this region, virtually all carnivores could predate, at least occasionally, on one or more forest‐grouse life stages. Therefore, we restricted this study to those that geographically or seasonally rely on forest grouse for prey or are perceived to impact forest‐grouse populations through predation. Respectively, these include golden eagle, eagle owl, northern goshawk and common buzzard (*Buteo buteo*) (Graham et al., 1995; Obuch & Bangjord, 2016; Reif et al., 2001; Tjernberg, 1981; Tornberg et al., 2009), and red fox, pine marten, least weasel (*Mustela nivalis*), stoat (*Mustela erminea*), and corvids (e.g., hooded crow *Corvus cornix*, rook *Corvus frugilegus*, raven *Corvus corax*, Eurasian jay *Garrulus glandarius*) (Baines et al., 2016; Fletcher et al., 2013; Kämmerle et al., 2017; Kurki et al., 1997; Saniga, 2002; Summers et al., 2004; Wegge & Kastdalen, 2007). Additionally, Eurasian lynx, owls (e.g., long‐eared owl *Asio otus*, tawny owl *Strix aluco*) and diurnal raptors (e.g., common kestrel *Falco tinnunculus*, Eurasian hobby *Falco subbuteo*) were included despite scarce evidence of their predation of forest grouse, due to the rich evidence of their interactions with the main predators of forest grouse (e.g., Linnell et al., 1998; Petty et al., 2003). Hereafter, species or groups of species are referred to as taxa. Selected taxa are sympatric within the geographical area reviewed with some exceptions where the largest predators (lynx, golden eagle, and eagle owl) remain absent in parts of central and western Europe and the British Isles (IUCN, 2020). Note that where pine martens co‐occur with the stone marten (*Martes foina*), their remains in the diet or kills of other predators are often indistinguishable. Thus, accounts of *Martes sp*. were included and reported where relevant.

Eurasian badgers (*Meles meles*) were initially part of this review as they can predate on forest grouse (Hounsome & Delahay, 2005; Jahren, 2012) and interact with other predators (Palomares & Caro, 1999). However, accounts of these interactions were rare in the literature (e.g., Sidorovich, 2011), and some accounts indicate suppression between foxes and badgers may be reciprocal suggesting that it occurs through competitive mechanisms and not predator interactions (Marcström et al., 1990 as cited by Selås, 1998; Trewby et al., 2008). Moreover, these interactions co‐occurred with facilitative interactions (Kowalczyk et al., 2008; Mori et al., 2015). Therefore, we excluded badgers from this review. Nonetheless, other instances of competitive or otherwise nonlethal interactions are described in the results and/or later discussed where they may contribute to elucidate the role of predator interactions as the drivers of population suppression. Large mammalian predators such as gray wolves (*Canis lupus*) or brown bears (*Ursus arctos*) may predate on forest grouse or interact with smaller predators. However, to the best of our knowledge there are no studies in Europe documenting ecologically meaningful interactions between them and grouse, or between brown bears and the predators listed above. Additionally, while three studies explored the relationship between wolves and red foxes in Europe (Elmhagen & Rushton, 2007; Pasanen‐Mortensen et al., 2013; Wikenros et al., 2017), these found either no effect, were confounded with the effect of lynx, or could be ascribed to a local (and possibly temporary) displacement rather than population suppression. Thus, these two large predators were also excluded from this study. Similarly, invasive species such as American mink (*Neovison neovison*) or raccoon dog (*Nyctereutes procyonoides*), or domestic animals such as dogs (*Canis familiaris*) or cats (*Felis catus*) were not included due to the scarcity of evidence of their interactions with forest grouse or native predators. Furthermore, their ecology may be influenced by factors that could obscure the underlying mechanisms of predator interactions (e.g., behavioral release, food sources unavailable to wild predators). Finally, we have also ignored intraspecies interactions (i.e., cannibalism).

We retrieved articles, book chapters, and reports published up to July 2019 and available online that addressed interactions among forest‐grouse predators in Europe. We used electronic database search engines (Web of Science, Google Scholar, ResearchGate) for two searches. The first sought to review predator's diet and detect instances of intraguild predation, while the second sought to document the strength and impact of such interactions. The first was structured as “predator AND diet” using the following search terms for predator: “lynx”, “red fox”, “golden eagle”, “eagle owl”, “goshawk”, “buzzard” and “pine marten”. The diet of the smallest predator taxa (diurnal raptors, owls, corvids, stoats, and weasels) was not reviewed but instances of intraguild predation by these predators were included if available. The second search was structured as “predatorA AND predatorB AND type of interaction”, where the type of interaction was a combination of: “intraguild”, “predation”, “killing”, “suppression”, “impact”. Additionally, the terms: “stoat”, “weasel”, “mustelid”, “corvid”, “owl”, “raptor” were added to the list of predators. Articles were first appraised from the title and abstract to include data on diet, killing, or population impacts of our predators of interest and within the geographical boundaries of this study. Reference lists of retrieved articles were screened for additional studies. Where a study was cited by another but itself not available, the data were extracted if the study area, sample size, and method were reported. This yielded a total of 240 studies. Eighty were later discarded. Seventy‐nine were dietary studies that did not contain accounts of intraguild predation, focused on specific components of the diet (e.g., plants), were aggregated at a high taxonomic level (e.g., carnivora, passerines) or were already included in another study. The majority were of pine marten (36) and red fox (19) as well as three of both species. Eight were of common buzzard, the same number as golden eagle's, three of lynx and two of eagle owl. One other study by Björklund et al. (2016), which addressed the relationship of northern goshawks and common buzzards in Finland, was excluded because it considered a migratory population of buzzards (unlike in the rest of Europe). Hence, the study did not report demographic effects. CNW and LR performed the literature search.

Results from diet studies of birds of prey are usually presented as the proportion of prey items belonging to a particular species relative to the total number of prey items across samples, while for mammalian predators these are generally presented as the proportion of samples (scats, kills, stomachs) with a given prey category in them. Occasionally, avian diet was quantified in the latter manner. We refer to these as Frequency of Occurrence (% FO, hereafter) irrespective of the method used. Dietary studies often report data aggregated at different arbitrary levels (e.g., nests, territories, regions, seasons, multiannual periods). Where available, the total frequency of occurrence per study was extracted from the papers or calculated from the data provided. To summarize the data, we averaged the frequency of occurrence of a given intraguild prey in the diet of an intraguild predator by method, weighing by the sample size of each study. For this, we only included papers with nonzero occurrence for a given species pair. Note that dietary data does not allow discerning predation from scavenging. The data are presented per species pair and, where relevant, by method. Multiple species pairs were grouped in single sections were deemed reasonable (e.g., similarities in the species involved). For each species pair, dietary data are presented first, followed by killings, annual killing rates, and any evidence of impacts on demographic parameters (i.e., breeding success, mortality). Evidence of population‐level effects, direct or indirect, is presented last and qualitatively evaluated as the suppression or, in their absence release, of a predator's abundance by another through lethal predator interactions. Interactions were deemed anecdotal when a single account was found across all the literature reviewed and empirical when there were two or more independent accounts.

## 3 RESULTS

**FIGURE 1:**
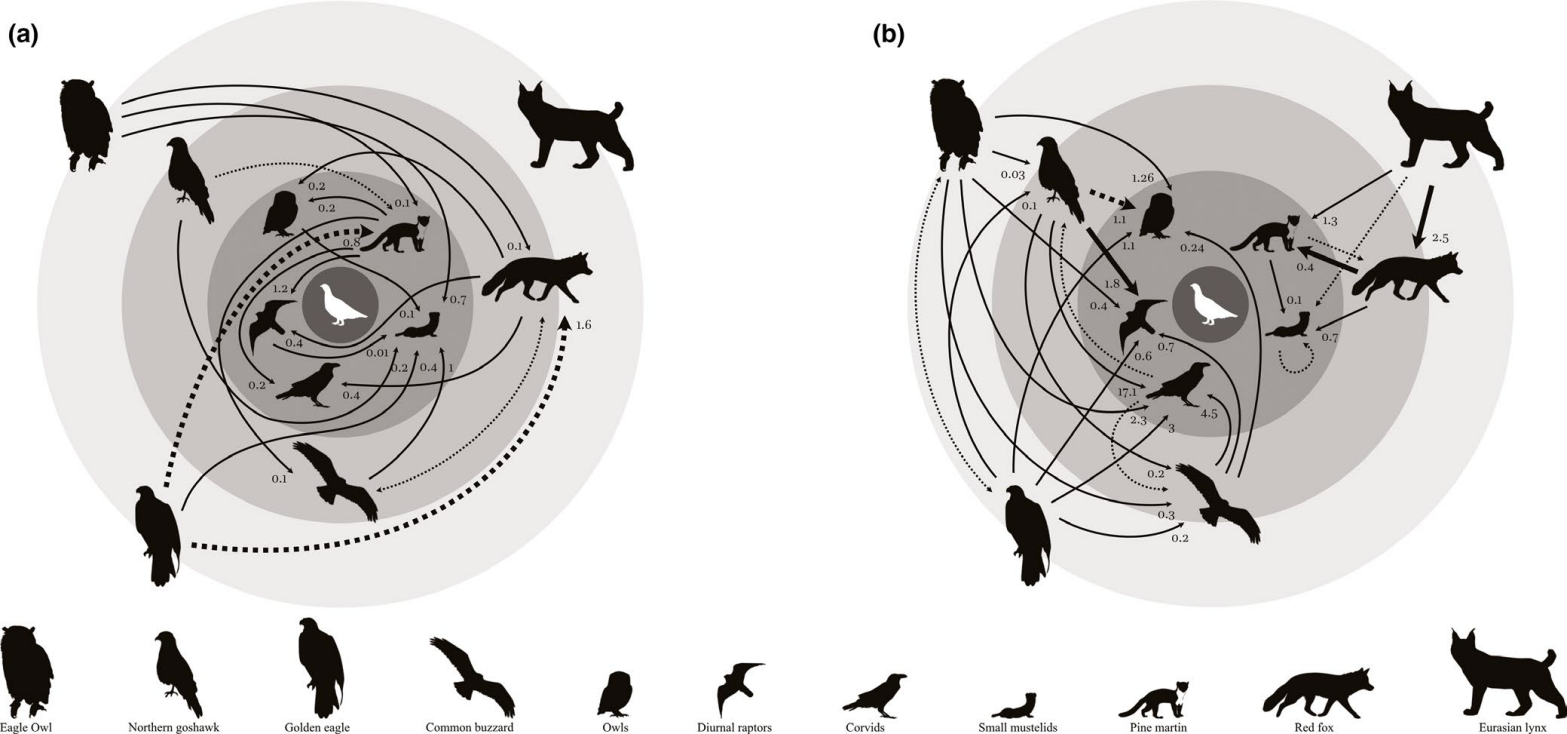
Diagram summarizing interactions among forest‐grouse predators. (a) Interactions between birds and mammals. (b) Interactions among birds and among mammals. Dotted lines (12) represent interactions based on anecdotal evidence. Solid thin lines (38) interactions based on at least two independent accounts. Solid thick lines (3) represent interactions with evidence of population suppression. Thick dotted lines (3) represent interactions with evidence of no population suppression. The direction of the arrows depicts the relationship from killer to victim. Numbers next to arrowheads represent the average frequency of occurrence (FO) weighted by sample size for the method with the largest sample size for each pair. For visual purposes, least weasels and stoats are combined (i.e., lines represent interactions with one or both small mustelids) and where applicable their FOs combined. All silhouettes were obtained from http://phylopic.org/, see Table S5 for full list of credits. Designed by Susanna Quer

A total of 160 relevant studies were compiled in this review (see Appendix S1 for the complete list and Figure S6 for geographical distribution), of which 130 contained diet data (Table S1), 16 presented information on the number of predators killed, annual killing rates or annual mortality (Table S2), and 24 reported data on suppression, release, or facilitation between predators (Table S3). By taxa, 27 studies were compiled that reported data on golden eagles as the killer predator, 26 and 23 on northern goshawks and eagle owls, respectively, 22 on Eurasian lynx, and 21 on common buzzards. Sixteen studies also included data on red foxes, 14 on pine martens, and 11 on owls as killers. Only two studies contained data on corvids and diurnal raptors as killers, and one on stoats. Forty‐four interactions between our 12 focal taxa were well documented in the literature, with an additional 12 supported by anecdotal evidence (i.e., single reports). Seventeen were between bird predators, five between mammal predators and 22 between birds and mammals. The most common intraguild predators were golden eagles and eagle owls accounting for 20.5% of the interactions each as they kill all other predators except lynx and each other only anecdotally (Table 1). Weasels were the most common intraguild prey as they fall victim to eight other predators (18.2% of interactions) followed by corvids, owls, and diurnal raptors with six killers (13.6%). Common buzzards and pine martens commonly interacted. They were frequent killers with 11.4% of interactions each. However, they both fell prey to four (9.1%) other predators. Next were goshawks and foxes. The former killed six (13.6%) taxa and was victim only to the two (4.5%) largest raptors: eagle owls and golden eagles. Foxes killed five (11.4%) other predators and were killed by three (6.8%), the two largest raptors and lynx. Lynx interacted with the least number of taxa. They were not killed by any of the other predators considered in this review and killed only foxes and martens. Thus, they were only reported in 4.5% of interactions. The grand mean of frequency of occurrence in the diet of another predator across all pairs of interacting species and methods was relatively low at 1.7% but ranged widely between 0.002% and 36.2% FO. With, on average, 3.7 empirically documented interactions per taxa, the community of grouse predators in Europe is highly interconnected (Figure 1).

**TABLE 1:** Number of focal taxa each predator kills or is victim to, and number of independent studies where they were reported as such (Killer/Victim). Multiple studies may report the same pairwise interaction. Numbers in parenthesis represent anecdotal interactions based on single accounts. Studies that reported no impact, facilitation or did not identify victims to species level were not included

Taxa	Killer	Victim	Total	Studies
Common buzzard	5(1)	4(2)	9	21/30
Eagle owl	9(1)	0(1)	9	23/1
Golden eagle	9(1)	0(1)	9	27/1
Pine marten	5(2)	4(1)	9	14/18
Least weasel	0	8(2)	8	0/46
Northern goshawk	6(1)	2(1)	8	26/14
Owls	2	6	8	11/53
Red fox	5(2)	3(2)	8	16/53
Diurnal raptors	1	6	7	2/43
Corvids	0(2)	6	6	2/75
Stoat	0(1)	5(2)	5	1/25
Eurasian lynx	2(1)	0	2	22/0

### 3.1 Raptors versus red fox and pine marten

Foxes are ubiquitous in the diet of golden eagles in Europe, detected in 22 out of the 26 diet studies with evidence of intraguild predation by eagles (Table 2). The bulk of the data comes from the analysis of pellets and prey remains with a 1.58% FO averaged across studies (*n* = 17,554 prey remains; Table 2). The largest estimates have been observed in the Swiss Alps at 9.5% FO (*n* = 21; Iselin & Hämmerle, 1960) and in the western Pyrenees at 11.9% FO (*n* = 235; Clouet et al., 2017) with relatively low sample sizes. Occasionally, foxes also fall prey to eagle owls (0.07% FO, detected in 10 studies, *n* = 91,135 prey remains; Table 2). A single instance of fox detected in the diet of common buzzards in Belarus does not amount to evidence of killing and is plausibly scavenging (0.4% FO, *n* = 1,065; Sidorovich, 2011). Pine martens are also predated by golden eagles, albeit less frequently than foxes; they were detected in seven golden eagle diet studies with an average 0.87% FO (*n* = 11,638 prey remains; Table 2). Pine martens were rare in the diet of eagle owls, detected in only two studies with an average of 0.1% FO (*n* = 19,842 prey remains; Table 2) and were found in a single instance in the diet of northern goshawks in Belarus (0.5% FO, *n* = 778 prey remains; Sidorovich, 2011).

**TABLE 2:** Summary table of intraguild predation (IGP). Frequency of Occurrence (% FO) is expressed as the weighted average across studies that include each pair of taxa and per method. Range provides the minimum and maximum IGP across studies for a given pair. Total sample size is added across studies

Killer	Victim	Frequency of Occurrence (% FO)	Range	Total Sample Size	Method	Studies x Pair	Studies x Killer	Total Studies
Common buzzard	Corvids	4.48	0.08–18.2	11,759	Analysis of pellets and prey remains	16	21	130
	Diurnal raptors	0.7	0.7–0.7	142	Analysis of pellets and prey remains	1		
	Least weasel	0.63	0.08–1.75	12,876	Analysis of pellets and prey remains	15		
	Owls	0.24	0.1–0.56	4,592	Analysis of pellets and prey remains	6		
	Red fox	0.4	0.4–0.4	1,065	Analysis of pellets and prey remains	1		
	Small mustelids	1.16	1.16–1.16	946	Analysis of pellets and prey remains	1		
	Stoat	0.32	0.12–0.54	1573	Analysis of pellets and prey remains	2		
Diurnal raptors	Least weasel	0.01	0.002–0.28	60,665	Analysis of pellets and prey remains	2	2	
Eagle owl	Common buzzard	0.32	0.04–1	39,812	Analysis of pellets and prey remains	9	20	
	Corvids	2.3	0.17–10.76	92,224	Analysis of pellets and prey remains	15		
	Diurnal raptors	0.39	0.09–2.1	89,367	Analysis of pellets and prey remains	13		
	Least weasel	0.44	0.07–4.7	95,744	Analysis of pellets and prey remains	11		
	*Martes* sp.	0.19	0.05–1.4	10,692	Analysis of pellets and prey remains	3		
	Mustelids	2.6	2.6–2.6	4,743	Analysis of pellets and prey remains	1		
	Northern goshawk	0.03	0.01–0.36	84,689	Analysis of pellets and prey remains	7		
	Owls	1.26	0.24–4.77	96,286	Analysis of pellets and prey remains	16		
	Pine marten	0.1	0.01–0.4	19,842	Analysis of pellets and prey remains	2		
	Red fox	0.07	0.02–0.31	92,135	Analysis of pellets and prey remains	10		
	Small mustelids	1.19	1.11–1.35	3,370	Analysis of pellets and prey remains	2		
	Stoat	0.25	0.09–5.5	93,808	Analysis of pellets and prey remains	11		
Eurasian lynx	Least weasel	3	3–3	33	Analysis of stomach contents	1	16	
	Mustelids	2.1	2.1–2.1	146	Analysis of stomach contents	1		
	Pine marten	1.34	0.8–2.84	1,292	Analysis of scats	3		
	Pine marten	0.58	0.1–2	923	Snow‐ or radio‐tracking lynx	3		
	Red fox	2.48	1.42–2.7	1,358	Analysis of scats	3		
	Red fox	2.49	0.8–7.1	1,126	Analysis of stomach contents	7		
	Red fox	7.6	7.6–7.6	66	Hunter interviews	1		
	Red fox	5.71	0.9–13	1,282	Snow‐ or radio‐tracking lynx	6		
Golden eagle	Common buzzard	0.21	0.01–7.7	11,129	Analysis of pellets and prey remains	5	26	
	Corvids	3.05	0.3–21.7	17,454	Analysis of pellets and prey remains	19		
	Corvids	0.6	0.6–0.6	181	Video recording prey deliveries	1		
	Diurnal raptors	0.8	0.8–0.8	120	Video recording prey deliveries	1		
	Diurnal raptors	0.57	0.14–9.36	15,651	Analysis of pellets and prey remains	15		
	Eagle owl	1.25	1.25–1.25	80	Analysis of pellets and prey remains	1		
	Least weasel	0.02	0.01–1.25	6,987	Analysis of pellets and prey remains	2		
	*Martes* sp.	3	3–3	235	Analysis of pellets and prey remains	1		
	Mustelids	2.52	1.3–10.1	3,993	Analysis of pellets and prey remains	9		
	Northern goshawk	0.13	0.1–0.21	9,710	Analysis of pellets and prey remains	2		
	Northern goshawk	0.6	0.6–0.6	181	Video recording prey deliveries	1		
	Owls	1.09	0.15–2.5	14,669	Analysis of pellets and prey remains	13		
	Owls	1.4	1.4–1.4	71	Video recording prey deliveries	1		
	Pine marten	0.87	0.6–1.81	11,638	Analysis of pellets and prey remains	6		
	Pine marten	1.7	1.7–1.7	120	Video recording prey deliveries	1		
	Red fox	1.58	0.15–11.9	17,554	Analysis of pellets and prey remains	20		
	Red fox	3.3	3.3–3.3	181	Video recording prey deliveries	1		
	Red fox	9.5	9.5–9.5	21	Visual observations of prey remains	1		
	Small mustelids	1.2	1.2–1.2	247	Analysis of pellets and prey remains	1		
	Stoat	0.35	0.1–0.94	11,233	Analysis of pellets and prey remains	4		
	Stoat	1.4	1.4–1.4	71	Video recording prey deliveries	1		
Northern goshawk	Common buzzard	0.16	0.03–0.9	13,895	Analysis of pellets and prey remains	6	20	
	Corvids	17.14	2.9–36.2	46,581	Analysis of pellets and prey remains	14		
	Corvids	26.7	26.7–26.7	146	Video recording prey deliveries	1		
	Diurnal raptors	1.8	0.1–4.17	34,799	Analysis of pellets and prey remains	7		
	Least weasel	0.16	0.04–0.8	20,020	Analysis of pellets and prey remains	6		
	Owls	1.07	0.12–2.16	39,234	Analysis of pellets and prey remains	9		
	Pine marten	0.5	0.5–0.5	778	Analysis of pellets and prey remains	1		
	Stoat	0.07	0.06–0.12	11,971	Analysis of pellets and prey remains	3		
Owls	Least weasel	0.05	0.01–0.9	242,551	Analysis of pellets and prey remains	11	11	
	Small mustelids	0.77	0.77–0.77	520	Analysis of pellets and prey remains	1		
	Stoat	0.02	0.01–0.17	77,123	Analysis of pellets and prey remains	6		
Pine marten	Common buzzard	0.07	0.06–0.1	2,589	Analysis of scats	2		
	Corvids	0.21	0.07–1.29	8,400	Analysis of scats	6		
	Corvids	1.56	1.56–1.56	450	Analysis of stomach contents	1		
	Diurnal raptors	1.22	0.26–1.63	1,305	Analysis of scats	2		
	Least weasel	0.09	0.09–0.1	7,235	Analysis of scats	2		
	Mustelids	0.12	0.12–0.12	1735	Analysis of scats	1		
	Owls	0.24	0.06–0.58	4,962	Analysis of scats	4		
	Raptors	0.1	0.1–0.1	854	Analysis of scats	1		
	Stoat	0.28	0.28–0.28	5,677	Analysis of scats	1		
Red fox	Corvids	0.35	0.1–1	1,389	Analysis of scats	2	15	
	Diurnal raptors	0.35	0.2–1.82	1,087	Analysis of scats	3		
	Least weasel	0.65	0.5–2	5,372	Analysis of scats	5		
	*Martes* sp.	0.7	0.7–0.7	285	Analysis of stomach contents	1		
	Medium mustelids	0.5	0.5–0.5	6,694	Analysis of scats	1		
	Owls	0.21	0.04–0.5	1,028	Analysis of scats	2		
	Pine marten	0.4	0.4–0.4	4,175	Analysis of scats	1		
	Pine marten	0.5	0.5–0.5	224	Analysis of stomach contents	1		
	Small mustelids	0.55	0.3–10	7,839	Analysis of scats	4		
	Stoat	0.02	0.02–0.02	4,175	Analysis of scats	1		

There is evidence of pine martens predating raptors. Owls were detected in their diets in four studies averaging 0.24% FO (*n* = 4,962 scats). So were diurnal raptors detected in two studies at 1.22% FO (*n* = 1,305 scats) and even common buzzards in another two (0.07% FO, *n* = 2,589 scats). Additionally, over an 8‐month radio‐tracking study by Sidorovich (2011), a single pine marten killed, and at least partly consumed, two adult Tengmalm's owls, one adult tawny owl and one long‐eared owl. Such predation on raptors is most likely the result of nest raids. From radio and snow tracking of pine martens, Sidorovich (2011) reported events of nest predation by pine marten on Eurasian hobby (*n* = 1), buzzard (*n* = 1), Ural owl (*n* = 2) and tawny owl (*n* = 4). In a study in south‐east Norway where owls were provided with nest boxes, martens predated 33% of hawk owl nests (*n* = 12) and 25.9% of Tengmalm's owl nests (*n* = 187; Sonerud, 1985). Marten predation on Tengmalm's owl nest boxes has been documented to reach up to 50% in the Czech Republic, which the authors attributed to low local structural complexity in the forest, combined with high densities of owl nest boxes (Zárybnická et al., 2015). There are fewer accounts of raptors predated by red foxes; yet newly fledged chicks may be vulnerable to terrestrial predators. Sunde (2005) reported that foxes were responsible for 18 out of 19 predated tawny owl fledglings where the predator could be identified. Sidorovich (2011) also described four instances where a radio‐tracked fox killed, and at least partly ate, one common buzzard, one Tengmalm's owl, one Ural owl and one tawny owl, but found no evidence of raptor prey in the diet of foxes despite a large sample size (*n* = 4,175 scats). Two other studies, however, detected diurnal raptors in the diet of fox at 0.27% FO (*n* = 1,032; Table 2) and another two detected owls at 0.21% FO (*n* = 1,028; Table 2).

The only study attempting to detect evidence of population suppression between raptors and medium‐sized mammals in Europe was done by Lyly et al. (2015) in Finland, combining 23 years of country‐wide data from nesting records of golden eagle (*n* = 6,569 records) and mesopredator track counts from the Finnish Wildlife Triangle Scheme (*n* = 17,808 records). Using generalized additive models, this study revealed a nonlinear relationship (*p* = .004; Table S3) whereby pine marten abundance was weakly depressed at high eagle territory densities (>1 territory/100 km^2^) from approximately 0.8 to ca. 0.6 tracks per day, but increased by a similar amount from low to intermediate eagle densities (ca. 0–1 territory/100 km^2^; Lyly et al., 2015). The same study found that red fox abundance was positively associated with eagle density (*p* = .021; Table S3) increasing by approximately 1.7 tracks per day as eagle territory density ranged from zero to four territories per 100 km^2^. In both cases, the authors attributed these positive relationships between golden eagles and red foxes and pine martens to environmental covariates that were not included in the analyses and reflected similar prey preferences (e.g., reindeer carrion, hares) or habitat (e.g., forest age, density).

To conclude, it is unclear how the varying contributions of foxes and martens to raptors’ diet translate to meaningful predation rates (i.e., number of fox/marten predated per raptor and year), and while the potential for population suppression by most raptors remains untested, the fact that eagles, with the highest occurrence of mammal carnivores in their diets, fail to do so, suggests that no single raptor can suppress fox or marten populations. On the other hand, it appears unlikely that marten and fox predation on adult or juvenile raptors or their nests are demographically significant, except in rare circumstances.

### 3.2 Raptors versus small mustelids

There is a large body of empirical evidence showing that weasels and stoats are “bite‐size” predators that fall prey to larger avian and mammalian predators (see Lambin, 2017). The most commonly reported interaction among raptors and small mustelids is between common buzzards and weasels (*n* = 15 studies; Table 2), with an average 0.63% FO (*n* = 12,876 prey remains) followed by eagle owls and weasels, and smaller owls and weasels (*n* = 11 studies each) at 0.44% FO (*n* = 95,744 prey remains) and 0.05% FO (*n* = 242,551 prey remains), respectively. The interactions of goshawks with weasels are relatively common, with six studies and an occurrence of 0.16% (*n* = 20,020 prey remains). Weasels are the least documented in the diets of golden eagles and diurnal raptors (*n* = 2 studies each) and frequencies of 0.02% (*n* = 6,987 prey remains) and 0.01% (*n* = 60,665 prey remains), respectively (Table 2). The most common interactions between large raptors and stoats is with eagle owls (*n* = 11 studies) at 0.25% FO (*n* = 93,808 prey remains), followed by smaller owls (*n* = 6 studies) with 0.02% FO (*n* = 77,123 prey remains) and golden eagles (*n* = 4 studies) at 0.35% FO (*n* = 11,233 prey remains; Table 2), though an additional study using video recordings at nests estimated an occurrence of stoat in the diet of golden eagles of 1.4% FO (*n* = 71 prey deliveries; Table 2). Three studies have documented stoats in the diet of goshawks with an average occurrence of 0.07% (*n* = 11,971 prey remains), but only two documented stoats in the diet of buzzards with 0.32% FO (*n* = 1,573 prey remains). Eagle owls hold the highest estimates of occurrence for both weasels (4.7% FO, *n* = 4,646; Sidorovich, 2011), in Belarus, and stoats (5.5% FO, *n* = 183; Emmett et al., 1972), in Sweden.

It has been suggested that predation by raptors may amount to a significant top‐down influence on small mustelid population dynamics, especially where snow cover is limited, and mustelids are exposed to predation year‐round (Lambin, 2017; Oksanen et al., 2001). In their study, Korpimäki and Norrdahl (1989) tentatively estimated that despite the low prevalence of weasels and stoats in the diet of birds of prey, predation by raptors could account for 20% and 80% of the small mustelids’ mortality during high and low vole‐abundance years, respectively. This highlights that the annual proportion of the small mustelids’ productivity killed could be demographically more significant than their proportional contribution to raptor diet. However, these estimates are contingent on the precision of the abundance estimates of weasel and stoat populations, which were based on crude snow‐track counts. Additionally, these counts were done before the mustelids’ breeding season and do not account for density‐dependent compensation of losses to predation. Rigorously quantifying the implications of such predation rates for small mustelid populations has been and remains beyond the resolution of available field techniques (Lambin, 2017). Based on the available evidence, we cannot conclude whether small mustelids are demographically suppressed by raptors or not.

### 3.3 Raptors versus corvids

Large raptors are known to prey on corvids throughout their range. For instance, corvids can be an important prey of northern goshawks. They were detected in 14 out of the 20 diet studies averaging 17.14% FO (*n* = 46,581 prey remains; Table 2) of the goshawk's diet. The highest figure (36.2% FO, *n* = 2,230 prey remains) was reported in the UK (Toyne, 1998). Their importance in goshawk diet varies seasonally and spatially. Tornberg et al. (2009) found that corvids increased from approximately 5% to more than 20% FO from spring to summer (*n* = 2,596 prey remains), as juveniles become available. Their occurrence in the diet was also correlated with the prevalence of agricultural habitats. Corvids also increased in the diet of goshawks as they became food limited. In a spruce plantation forest in England, the contribution of crows and rooks in the diet of goshawks increased from 11% to 19% FO (*n* = 7,763 prey items) as the density of goshawks increased and their main prey declined between 1973 and 2014 (Hoy et al., 2017).

Corvids are frequently predated by common buzzards too. We found 16 diet studies (out of 21) documenting corvid consumption, averaging 4.48% FO (*n* = 11,759 prey remains; Table 2), with the highest estimate also reported in the UK at 18.2% FO (*n* = 253 prey remains; Sim et al., 2001). Interestingly, one of the highest frequencies of occurrence reported (14.7% FO) is from an area where the buzzards’ main prey, microtine voles, are absent (Ireland; Rooney & Montgomery, 2013). Corvids have a similar importance in the diet of golden eagles, being detected in 20 studies with an average of 3.05% FO (*n* = 17,984 prey remains; Table 2). Remarkably, the five highest frequencies are all from French studies (Table S1), with the highest, in the western Pyrenees, reaching 21.7% FO (*n* = 235 prey remains; Clouet et al., 2017). We also found 15 studies of eagle owls that reported corvids in their diet, with an average occurrence of 2.3% (*n* = 92,224 prey remains).

Although corvids are subordinate species to large adult raptors, they are efficient nest predators. Byholm and Nikula (2007) attributed 14.9% of goshawk nest losses (*n* = 87) to corvid predation and Kostrzewa (1991) observed Eurasian jays predating two buzzard nests.

The varying contribution of corvids to the diet of raptors seems to reflect their availability relative to each raptor's main prey. However, we lack the evidence to confirm or reject whether predation by any one raptor may suppress the populations of corvids.

### 3.4 Golden eagle versus other forest dwelling raptors

Golden eagles are the largest raptor considered in this review, and smaller diurnal raptors and owls were frequently reported in their diet with 15 and 13 accounts, respectively. Their overall occurrence, however, is small with 0.57% FO (*n* = 15,651 prey remains) for diurnal raptors and 1.09% FO (*n* = 14,669 prey remains; Table 2) for owls. A remarkable outlier is from the western Pyrenees, where diurnal raptors comprised 9.4% FO of the eagle's diet owing to the large contribution of common buzzards (7.7% FO, *n* = 235; Clouet et al., 2017). Such a large proportion of buzzards constitutes another outlier as they were detected at an average 0.21% FO (*n* = 11,129 prey remains) across all five studies. Northern goshawks too can fall prey to golden eagles. We have found evidence of this in three studies, one based on video recordings of prey deliveries (*n* = 181) where a single goshawk was brought to the nest (0.6% FO; Skouen, 2012) and another two based on prey remains analysis averaging 0.13% FO (*n* = 9,710). Strikingly, an instance of golden eagle predation on eagle owls was reported in France (1.25% FO, *n* = 80 prey remains; Austruy & Cugnasse, 1981).

While the interactions between golden eagles and other raptors through intraguild predation are well documented, we found no evidence assessing the existence of population suppression of other forest predators in the boreal and temporal forests of Europe, and hence cannot conclude either way about its existence.

### 3.5 Eagle owl versus other raptors

Eagle owls are large nocturnal raptors with diverse diets that predate on a wide range of other birds of prey. Diurnal raptors consistently made up small fractions of the eagle owl's diet, being detected in 13 studies at 0.39% (*n* = 89,367 prey remains). Smaller owls were detected in 16 studies comprising a relatively larger proportion of the diet (1.26% FO, *n* = 96,286 prey remains; Table 2). Common buzzards occurred in their diets at a similar frequency as smaller raptors. They were detected in nine studies at an average 0.32% FO (*n* = 39,812 prey remains). In contrast, goshawks comprised only 0.03% FO (*n* = 84,689 prey remains; Table 2) across seven studies. One study also reported eagle owls killing two golden eagle adults in Belarus (Sidorovich, 2011).

While no studies have attempted to estimate the proportion of the population of a given raptor species that may be removed by eagle owls, Mueller et al. (2016) detected a strong change in nest site selection by goshawks after the recolonization of the eagle owl in the Westphalia (Germany), as well as an increase in brood failure in both goshawk (10% increase, *n* = 427 goshawk breeding attempts) and common buzzard (5% increase, *n* = 2,252 buzzard breeding attempts). In the same region, Chakarov and Krüger (2010) also reported an increase in brood failure for both goshawks (>50%, *n* = 355 goshawk breeding attempts) and buzzards (ca. 20%, *n* = 1,504 buzzard breeding attempts) as well as an overall decrease in (standardized) reproductive success in response to the large owl (of −1.2 for goshawks and −0.525 for buzzards). Additionally, in northern Germany, where both species were monitored between 1975 and 2002, Busche et al. (2004) reported eagle owls to be responsible for up to 26% of the goshawk nest losses (*n* = 51). Furthermore, 22 out of the 37 nests used by eagle owls in this study were originally used by goshawks and another 11 were originally buzzard nests. Similarly, in Finland, 11.5% (*n* = 87) of goshawks nest losses were attributed to predation of nestlings by eagle owls (Byholm & Nikula, 2007). The effect of eagle owls on smaller owls has been addressed in only two studies. In the Italian Alps, Sergio et al. (2007) found, using regression analysis, that the probability of a nest site being selected by tawny owls increased with distance to eagle owl nests at intermediate eagle owl densities (1–2 territories per 100 km^2^; 0.05 ± 0.01, *p* < .02, *n* = 32 eagle owl territories and 36 tawny owl territories; Table S3). Additionally, at high eagle owl densities (3 territories per 100 km^2^), tawny owls avoided the preferred hunting habitat of eagle owls altogether (0.2 ± 0.07, *p* < .05). Instead, the laying date (*t* = 0.31, *df* = 17, *p* > .05), clutch size (*t* = 0.61, *df* = 19, *p* > .05), and number of fledglings (*t* = 1.43, *df* = 18, *p* > .05) of Tengmalm's owls were unaffected by the presence of eagle owls (*n* = 20 nest boxes in eagle owl territories and 20 in their absence; Hakkarainen & Korpimaki, 1996).

The evidence regarding potential population‐level suppression of large raptors by eagle owls is mixed. In western Germany, Chakarov and Krüger (2010) reported a ca. 23% decline of goshawk breeding densities, from approximately eight to six goshawk pairs per 100 km^2^ following the recolonization of eagle owls, while goshawk densities increased in two neighboring study sites where the owls had not yet arrived. Similarly, Busche et al. (2004) reported a negative correlation between the breeding densities of both raptors (*r* = −0.57, *p* < .01, *n* = 180 goshawk and 42 eagle owl breeding attempts) in northern Germany where following the reintroduction of eagle owls, goshawk numbers declined from 18 to 6 breeding pairs. However, in a reanalysis of Chakarov and Krüger's (2010) data, pooling across the three study sites and with four years of additional data, Mueller et al. (2016) did not detect any decline in goshawk breeding numbers after the return of eagle owls despite changes in nest site selection and increased brood failure. Instead, both species increased in abundance. The evidence of smaller owl suppression is similarly mixed. The breeding densities of tawny owls were negatively correlated with eagle owl's (*r* = −0.79, *p* = .027). Tawny owl densities decreased from approximately 45 territories per 100 km^2^ in the absence of the larger owls down to ca. 12 territories per 100 km^2^ at high eagle owl densities (​3 territories per 100 km^2^; Sergio et al., 2007). On the other hand, Tengmalm's owls nest box occupancy was unchanged by the presence of eagle owls (χ^2^ = 0.2, *df* = 1, *p* = .66; Hakkarainen & Korpimaki, 1996). Nonetheless, Sergio et al. (2007) argue that the suppression of a dominant competitor, tawny owls, could benefit other species, which could include Tengmalm's owls, as reflected by the positive correlation between owl assemblage diversity and eagle owl density (*r* = 0.9, *df* = 10, *p* = .03).

Intraguild predation of other raptors, even of large species, as well as impacts on their breeding performance are well documented. Evidence of population suppression, however, is mixed, and the relative importance of direct (predation, killing) and indirect (avoidance) predator interactions is uncertain. If present, population suppression may require the combination of both.

### 3.6 Northern goshawk versus other raptors

Goshawks are large birds of prey known to predate on smaller raptors. Across Europe, diurnal raptors and owls are well documented in their diets, present in seven and nine studies and comprising 1.8% (*n* = 34,799 prey remains) and 1.07% FO (*n* = 39,234 prey remains; Table 2), respectively. Common buzzard occurrence was also documented in six studies at an average frequency of 0.16% FO (*n* = 13,895 prey remains; Table 2). A crucial finding was the per capita increase in the frequency of occurrence of small raptors in the diet of goshawks from 2% to 8% FO (*n* = 7,763 prey remains) in Kielder Forest, England (Hoy et al., 2017), as the densities of goshawks increased and their main prey species (pigeons and game birds) declined over 41 years. The authors attributed this to increasing levels of food limitation. Kestrels were the main victim, accounting for nearly 50% of all raptors consumed, followed by tawny owls (23%) and sparrowhawks (10%).

Goshawks can depress other raptors’ breeding success. In Westphalia, Germany, Kostrzewa (1991) found that buzzard breeding success was positively correlated with increasing distance to goshawk nests (*r* = 0.28, *p* < .0001, *n* = 282 buzzard breeding attempts). In another study in the same region, Krüger (2004) documented a negative effect of goshawk hunting activity on buzzard breeding success (−0.577 ± 0.102, *p* = .001, *n* = 106 breeding attempts). These results were later confirmed by Mueller et al. (2016) using a long‐term dataset where increasing distance to goshawk nests led to higher buzzard breeding success (0.04 ± 0.02, *p* = .038, *n* = 2,252 buzzard and 427 goshawk breeding attempts). In Ostrobothnia, Finland, Hakkarainen et al. (2004) found that the production of fledglings by buzzards declined by 20% within 1 km of a goshawk nest when compared to those over 3 km away (from 2.02 ± 0.07 to 1.64 ± 1.8, *n* = 419 breeding attempts from 1983 to 1996). There are multiple instances of goshawk predating buzzards nests (five observed by Kostrzewa, 1991 and seven by Krüger, 2004) as well as taking over buzzard territories; 11% of buzzard territories (*n* = 119) were overtaken by goshawks in Germany (Kostrzewa, 1991) and in Finland 64% of goshawk territories (*n* = 28) were settled in previous buzzard territories (Hakkarainen et al., 2004). However, Krüger (2004) argued that lower breeding output was mostly due to adult buzzards abandoning their nests after being exposed to goshawks. Indeed, Hakkarainen et al. (2004) reported a fourfold probability of nest failure near goshawks. Furthermore, Krüger (2002) were able to induce a ca. 40% lower breeding success by exposing buzzard nests to playback calls of goshawks.

The demographic impact goshawks exert on their raptor prey has been well documented for small raptors and owls in the UK, with varying effects between victim species. Petty et al. (2003) argued that goshawks were responsible for the decline of kestrels, with 115 kestrels killed per year in an area that contained approximately seven breeding pairs. This implied that most of the predation occurred on the presettled component of the kestrel population. The same study also documented a decline of short‐eared owls concomitant with an increase in goshawk numbers over 23 years. In the same study area, Hoy et al. (2015) found that large losses of tawny owls (estimated at 72 and 159 owls per year when goshawks were at intermediate and high goshawk densities, respectively) did not translate to population‐level impacts because goshawks disproportionately killed juveniles and senescent individuals with low reproductive value. Only two studies referred to potential impacts of goshawks on other large raptors. Kostrzewa (1991) qualitatively observed that following the establishment of goshawks into new territories, local buzzard densities declined as they moved to other areas. Gryz and Krauze‐Gryz (2019) found a strongly negative correlation (*r* = −0.93, *p* < .0001) between breeding densities of buzzards and goshawks in a long‐term polish study (1982–2018), where buzzards increased by a factor of 2.2 after goshawks crashed.

The compiled evidence illustrates several points. First, the strength of intraguild interactions is likely to vary according to the availability of goshawk's main prey, such that dietary information alone cannot be used to infer the impact on the victim species. Second, suppression of small raptors by goshawks is possible, but its effectiveness may depend on the population component of the intraguild prey affected (e.g., breeding adults, young, or senescent individuals). Lastly, population‐level impacts on buzzards, if true, are seemingly mediated mostly by indirect interactions.

### 3.7 Buzzard versus other forest dwelling raptors

Common buzzards are the smallest of the large raptors considered in this review. They are also the least likely to predate on other raptors, with a single study, in the Italian alps, reporting the occurrence of diurnal raptors in their diet (0.7% FO, *n* = 142 prey remains; Sergio et al., 2002) though another study reported the killing of a diurnal raptor by a buzzard—a Eurasian hobby in Belarus (Sidorovich, 2011). Predation on owls is reported more frequently but remains a small portion of the studies with evidence of intraguild predation by buzzards (six out of 21; Table 2), and owls make only a small contribution to their diet (0.24% FO, *n* = 4,592 prey remains; Table 2). Accordingly, we found no evidence of buzzards removing significant proportions of their victim species population or exerting any form of population‐level suppression and do not expect them to do so.

### 3.8 Red fox versus pine marten

Foxes rarely predate (i.e., kill and consume) martens. Only two studies in Europe reported pine martens to occur in the diet of red foxes (Table 2): Kidawa and Kowalczyk (2011) analyzed 224 fox stomachs in Poland and reported one instance, resulting in a 0.5% FO of martens in the diet of foxes, while Sidorovich (2011) analyzed 4,175 scats in Belarus and detected them at 0.4% FO. Two other studies reported the occurrence of *Martes* sp. and medium‐sized mustelids in Denmark (0.7% FO, *n* = 285 fox stomachs; Pagh et al., 2015) and Belarus (0.5% FO, *n* = 6,694 fox prey items; Sidorovich et al., 2006). There are, however, additional instances of foxes killing pine martens. Lindström et al. (1995) compiled 16 instances from the literature where pine martens (14 adults and 2 juveniles) were killed by red foxes in Scandinavia. The same study reported two pine martens killed by foxes out of 26 radio‐tracked individuals in Sweden and Norway. In Belarus, radio and snow‐tracked foxes killed two adult martens in winter and summer in addition to seven kits in summer (Sidorovich, 2011). The killed pine martens were not always consumed: the two martens reported in Lindström et al. (1995) were found buried and uneaten, and in Belarus, only three out of the 11 martens killed were eaten (73% uneaten; Sidorovich, 2011). A single case of reversed intraguild predation (i.e., pine marten killing red fox cubs) has been observed in a mixed‐pine forest of north‐east Poland (Brzeziński et al., 2014).

A single estimate of the proportion of pine marten removed from the population by red foxes is available from the literature. Based on two mortality events after radio‐tracking 16 pine martens in Grimsö (Sweden), seven in Varaldskogen (SE Norway), and three near Trondheim (Norway) over seven years, Lindström et al. (1995) extrapolated that 13% of the adult population of pine martens was lost annually to foxes.

Evidence gathered during the epizootic sarcoptic mange (*Sarcoptes scabiei*) outbreak that markedly reduced red fox populations in the late 1970s and 1980s across Fennoscandia supports the occurrence of population‐level suppression of pine marten by red fox. Based on 6,800 annual questionnaires to active hunters between 1971 and 1993, the harvest rates of pine martens in Norway were estimated to have increase fivefold following the decline of red foxes (Smedshaug et al., 1999), with a significant negative correlation in the harvest rates of both species (*r* = −0.55, *p* = .0002). A similar figure was obtained by Lindström et al. (1995) in Sweden, where the number of pine marten tracks in the snow increased by a factor of 5.4 after the decline of foxes (*p* = .011, *n* = 12 km of annual snow tracking in 1974–1980 and 1990–1993). The same study also reported a negative correlation between the number of harvested red foxes and pine martens in 23 of the 24 counties of Sweden (*r* = −0.88 *p* < .05). In Finland, contrastingly, Kurki et al. (1997) found a positive correlation between snow‐track counts of both species (*r* = 0.56, *p* < .05; *n* = 1,500 wildlife triangles sampled biannually; 1989–1994). However, a later study using additional data up to 2011 and generalized additive models, found a significant nonlinear relationship (*p* < .001) between fox and marten track counts, such that pine marten tracks per day increased from ca. 0.5 to 0.9 as red fox increased from zero to approximately 100 tracks per day, after which the pine marten index dropped to approximately 0.15 as red fox increased to over 200 tracks per day (Lyly et al., 2015).

The above findings have been interpreted as reflecting competition for food and habitat (Lindström, 1989; Storch et al., 1990). This was tested by Storch et al. (1990) in Grimsö, Sweden, by snow‐tracking pine martens over two winters before the collapse of foxes (totaling 126 km) and for one winter after the collapse (76 km). Additionally, three pine martens were radio‐tracked after the collapse. Pine marten habitat use remained the same before and after the decline of foxes, and between the two tracking methods, suggesting that marten habitat use was not restricted by competition from foxes. Alternatively, red foxes and pine martens might compete over deer carcasses during winter, as the frequency of ungulate carrion in marten winter scats increased by over 30% following the decline of foxes. This was based, however, on a relatively small sample size (before *n* = 51, after *n* = 43; Storch et al., 1990). Furthermore, Wikenros et al. (2014) found that neither the frequency of visits by pine marten to wolf‐killed moose carcasses nor the time spent vigilant at these sites were influenced by fox activity, but instead were determined mainly by small‐scale plant cover and vegetation structure.

To summarize, despite small sample sizes, the above studies provide good support for foxes regularly engaging in interspecific killing and occasional predation of pine martens. Additionally, the evidence illustrates how the strength of the interaction could be severely underestimated if based on consumption alone given the small proportion of consumed intraguild prey. Finally, the consistent pattern of pine marten abundance increasing in response to a decline in fox numbers detected both with snow tracking and bounty data in two countries and at multiple scales indicates that pine marten populations can be limited by red foxes. While it is not possible to discern the relative importance of different types of interactions, the above evidence suggests that predator interactions are a significant contributing factor.

### 3.9 Red fox and pine marten versus small mustelids

Small mustelids fall prey not only to avian predators but terrestrial predators as well. While stoats were detected in the diet of foxes in a single study from Belarus (Sidorovich, 2011) and at a low frequency (0.02% FO, *n* = 4,175 scats), weasels were found as fox prey in five studies with an average occurrence of 0.65% FO (*n* = 5,372 scats; Table 2). An additional three studies reported the occurrence of both species of small mustelids with an average of 0.59% FO (*n* = 1661 scats; Table 2). Dell’Arte et al. (2007) reported the highest estimate available at 10% FO based, however, on a small sample size (*n* = 58 scats). Weasels and stoats have been reported in the diet of pine martens in two and one studies, respectively. The former was found at a 0.09% FO (*n* = 7,235 scats) and the latter at 0.28% FO (*n* = 5,677 scats).

No study has estimated the proportion of the mustelid's population killed by foxes; yet this proportion should be larger than implied by contribution to diet alone given that foxes do not always eat the mustelids they kill (Latham, 1952). Sidorovich (2011) documented 54 weasels and two stoats killed by foxes of which only three weasels were eaten (94.4% and 100% uneaten, respectively). The same author also found eight weasels killed by pine marten, none of which were consumed. Thus far, the use of conventional diet analyses has prevented the detection of rare predation events and the identification of prey at the species level, but even if accurately estimated, occurrence in the diet would not be representative of the strength of the predator interaction, which could be underestimated by a factor of up to 17 (3:51 eaten versus uneaten weasels by fox; Sidorovich, 2011). Thus, despite potentially large killing rates of small mustelids by red foxes, in the absence of additional evidence, we remain unable to conclude whether predation by red foxes could suppress small mustelid populations or not. We do not expect pine martens to impose population suppression on the smaller mustelids.

### 3.10 Pine marten and red fox versus corvids

Pine martens are semiarboreal predators and can access birds in their nests. Foxes while strictly ground dwelling may occasionally predate on exposed birds. Corvids were detected in the diet of martens in more than half the studies reviewed (seven out of 11). Their average occurrence was 0.21% FO across the six studies based on scat analysis (*n* = 8,400 scats; Table 2). The highest occurrence was reported in Poland at 1.29% FO (*n* = 155 scats; Posłuszny et al., 2007). A single study of marten stomach contents from Sweden produced a similarly high estimate of 1.56% FO (*n* = 450; Helldin, 2000). Corvid predation by foxes was documented in two studies (0.35% FO, *n* = 1,389 scats; Table 2), but scavenging cannot be discarded. No studies have addressed the potential impacts of these terrestrial predators on corvids in Europe, but we do not expect them to occur.

### 3.11 Eurasian lynx versus red fox and pine marten

The Eurasian lynx is a large carnivore known to kill red foxes and pine martens, with which it coexists throughout much of its range, although the lynx is presently absent from most of the fox and marten's ranges in Europe. The prevalence of red fox in the diet of the Eurasian lynx has been well documented across boreal and temperate Europe. Seven studies found red fox remains in the contents of lynx stomachs with an average occurrence of 2.49% (*n* = 1,126; Table 2). A similar estimate of 2.48% FO (*n* = 1,358; Table 2) was found across three studies using scat analysis. The highest estimates typically come from studies with small sample sizes. These are 7.1% (*n* = 127) and 7% FO (*n* = 46) from Estonia (Valdmann et al., 2005) and Sweden (Liberg, 1997 as cited by Linnell et al., 1998), respectively, based on stomach content analyses and 2.7% FO (n = 1,024) from Belarus based on scat analysis (Sidorovich (2011). Interestingly, the average frequency of occurrence of fox in lynx kills, based on seven studies, was twice as high (5.71% FO, *n* = 1,282 kills; Table 2) compared with scat or stomach analyses, and the highest figure, from Switzerland, stood at 13% FO (*n* = 194 kills; Capt et al., 1993 as cited by Linnell et al., 1998). This may indicate that not all killed foxes are consumed. Indeed, in Norway, Sunde et al. (1999) compiled 19 instances of lynx predation on red fox, of which 7 were left uneaten (37%), and in Belarus, only nine out of 38 foxes killed by lynx in winter were partly eaten and none of the nine foxes killed during summer were consumed at all (81% uneaten; Sidorovich, 2011). Contrastingly, the eight foxes killed by lynx in Norway and Sweden reported by Linnell et al. (1998) were fully consumed.

In addition to prevalence of foxes in the diet of lynx, predation rates have been reported in several studies. Using data from a 10‐year study by Molinari‐Jobin et al. (2002), radio‐tracking 29 lynx in the Swiss Jura Mountains, Helldin et al. (2006) estimated an average annual kill rate across sex and age classes of 4.8 foxes per lynx and year. In the Swedish taiga, the annual killing rate of red foxes by lynx was estimated at 1.3 foxes per lynx based on snow tracking (8,339 km tracked between 1995 and 2001; Helldin et al., 2006). In the same study, the annual mortality of fox caused by lynx was estimated at 4% and 14% when snow‐ and radio‐tracking foxes (*n* = 20 radio‐tracked foxes), respectively. Additionally, using data provided by Sunde et al. (2000), Helldin et al. (2006) extrapolated an annual killing rate of 2.8 foxes per lynx and year in central Norway based on a single fox killed in a 4‐year radio‐tracking study of 11 lynx. A larger killing rate was estimated in Belarus, at 23 foxes per lynx and year (1,800 km of snow‐tracking data; 1990 to 2008; Sidorovich, 2011).

The interactions between Eurasian lynx and pine martens are less documented than with red foxes. Only three studies found marten remains in the scats of lynx, averaging 1.34% FO (*n* = 1,292 scats), with the highest of the three reported in Norway by Sunde et al. (2000) at 2.84% FO (*n* = 141). Additionally, Linnell et al. (1998) observed a lynx killing a pine marten and Jobin et al. (2000) reported a single pine marten killed by lynx (*n* = 617 kills). Okarma et al. (1997) also detected two instances of predation by lynx on pine martens (*n* = 172 kills) when radio‐tracking 18 lynx in Białowieża between 1991 and 1996, and Dunker (1988) (as cited by Linnell et al., 1998) reported three pine martens killed by snow‐tracked lynx (*n* = 127 kills). The only annual killing rate estimate available is from Belarus (1,800 km snow‐tracking 1990–2008) at nine martens per year per lynx based on a total of nine instances (only two with partial consumption; 78% uneaten; Sidorovich, 2011).

The population‐level impacts arising from intraguild interactions between lynx and fox have received considerable attention. Helldin et al. (2006) argued that a 10.5% annual decline in the local population of foxes in Grimsö, Sweden, based on the number of active dens (*n* = 200 dens checked annually, 1996‐2004), was at least partly explained by the recolonization of lynx. Using historical bounty data (from 1828 to 1917) across Sweden, Elmhagen and Rushton (2007) found that the combined indices of lynx and wolf were negatively correlated with fox indices (*r* = −0.55, *p* < .01). Similarly, using wildlife triangles data across Finland (*n* = 800–900 triangles per year, 1989–2005), Elmhagen et al. (2010) found the indices of lynx and fox to be negatively correlated both spatially and temporally (*r* = −0.28, *p* < .05; *r* = −0.33, *p* < .05, respectively). Using the same wildlife triangle data from Finland and additional data from Sweden (*n* = 163 wildlife triangles per year, 2001–2003) in a regression analysis based on generalized least squares, Pasanen‐Mortensen et al. (2017) confirmed the negative relationship between the density of fox and lynx (*p* < .0001; Table S3). In a meta‐analysis of red‐fox densities across Eurasia since the 1950s (*n* = 110 density estimates) and using Structural Equation Models, Pasanen‐Mortensen et al. (2017) determined that across the continent, lynx presence had a negative effect on fox densities (path coefficient = −0.77). Within the lynx's range, fox densities were lower by a factor of 10 (0.74:0.073), and 40% more variable (Robust coefficient variation 130:184) than outwith its range.

While at a large scale, the relationship between fox and lynx is reportedly negative, at a smaller scale, the presence of lynx has been positively associated with fox. For instance, using only the Swedish wildlife triangle data (*n* = 165, 2001–2003), Wikenros et al. (2017) found a positive association between the two predators (0.089 ± 0.028, *p* = not reported). Additionally, Helldin and Danielsson (2007) documented a 21.2% FO average increase in roe deer in the winter diet of fox after the recolonization of the lynx to Sweden (*n* = 680 scats).

To conclude, the evidence that the Eurasian lynx can suppress red fox populations through intraguild predation and interspecific killing is among the most compelling found in this review. Additionally, we see that suppression may be scale dependent, perhaps due to the provisioning of ungulate carcasses by lynx. There is no evidence to assess the relationship between Eurasian lynx and pine marten populations.

## 4 DISCUSSION

From our review of lethal interactions among predators of European forest grouse emerges a picture of a strongly interconnected community with 44 empirically documented pairwise interactions between the 12 taxa reviewed (Figure 1). We found mammals engaged in both killings with and without consumption of the victim (i.e., intraguild predation and interspecific killing, respectively), while we found no evidence of interspecific killing but of predation between avian predators. We only found compelling evidence that three pairwise predator interactions translated into population‐level suppression. Similarly, only three had strong evidence of no population suppression. This review highlights, however, that predators interact simultaneously with a large proportion of their guild's predators. Thus, focusing on potential cumulative effects of multiple interactions between predators may reveal additional instances of suppression. We identified two such possible instances accounting for 18 out of 38 undetermined interactions (i.e., interactions lacking evidence to conclude whether they incur in population suppression or not). We posit that interactions causing population suppression affect age classes of the victim population whose survival has large impact on the population growth rate, such as breeding adults in long‐lived species. Furthermore, such interactions need to occur at a sufficiently high frequency, which we argue could be motivated by food limitations and, in mammals, by competition for ungulate carcasses. Finally, this review also highlights the large uncertainty that predator interactions introduce to conservation practices of vulnerable prey species that focus on the management of predator abundances.

We collated evidence on (i) the occurrence of predators in the diet of other predators and (ii) in a few instances, the proportion of the victim population removed, annual killing or mortality rates, and (iii) on population‐level effects of intraguild interactions. Evidence was collated as reported in the literature, with the authors’ interpretations where relevant. Unavoidably, not all studies could be accessed, particularly older and non‐English articles and reports. Additionally, the widespread reliance upon prey remains collected from raptor nests and different research traditions certainly led to seasonal and geographic biases. Similarly, a publication bias toward unusual findings could inflate estimates of intraguild predation (e.g., Dell’Arte et al., 2007). In contrast, the potentially large proportions of unconsumed victims by mammalian predators could underestimate them. Extrapolating the evidence available, intraguild predation could be underestimated by 37%–81% and 78% in the case of red foxes and pine martens, respectively, in the diet of lynx, by 73%–100% and 94%–100% in the case of pine martens and small mustelids, respectively, in the diet of foxes, and small mustelids could go unnoticed in the diet of martens. Nonetheless, the studies reviewed here, particularly diet studies, are well distributed throughout the British Isles, Fennoscandia, Eastern Europe, and the Mountain systems of central (and south‐central) Europe (Appendix S1: Figure S6). Additionally, the large sample sizes typically achieved for most pairs of interactions (Table 2) and the weighted method to summarize the evidence would dampen the effects of unusually large figures based on small sample sizes. Certainly, our estimates of intraguild predation concur with previous reviews (e.g., Lourenço et al., 2011; Watson, 1998). More importantly, this review considers evidence of intraguild predation alongside evidence of killings without consumption, killing and mortality rates, and the evidence of population suppression of victims, thus offering a broadly representative view of the existence predator interactions in Boreal and Temperate Europe.

Evidence of the strength and impacts of predator interactions in the European literature remains scarce, not least due to the challenges of quantifying them in natural settings. Interactions are rare and elusive, and existing field techniques such as telemetry are costly and often provide limited sample sizes. Additionally, performing replicated experiments manipulating predator numbers at meaningful scales is often impossible. The resulting scarcity of evidence provides a limited scope for strong inference. However, a few highly detailed long‐term and/or geographically extensive case studies, often taking advantage of natural experiments of changes in predator abundance, offer crucial contrasts between the interactions of several taxa (e.g., Hoy et al., 2017; Hoy et al., 2015; Lyly et al., 2015; Petty et al., 2003). These are used below to formulate a general hypothesis of the conditions for population suppression through predator interactions. While this hypothesis as well as other inferences made require further validation, they are well founded in ecological theory and in line with the evidence reviewed here and the wider scientific literature, and we present them alongside potential methods and experimental designs that would contribute to test such inferences.

### 4.1 Predator interactions are the norm

Out of a total of 56 pairwise interactions among forest‐grouse predators detected in this review, we found empirical support (i.e., more than one account) for 44. All but the smallest taxa, the least weasel, engaged in predator interactions with other grouse predators at least anecdotally. Additionally, all species were on either the giving or the receiving end of lethal interactions simultaneously with multiple predators. This high degree of trophic connectivity is consistent with the view that interactions among predators are ubiquitous across continents and taxa (Arim & Marquet, 2004). As in previous studies, interactions between pairs of species were mostly unidirectional (Donadio & Buskirk, 2006; Palomares & Caro, 1999), with only five anecdotal instances of reciprocity (golden eagle and eagle owl, corvids and goshawks and buzzards, foxes and buzzards and martens and foxes). Moreover, three of these were adults of the “usual” victim killing young of the “usual” killer (corvids predating raptors nests and martens killing fox cubs). Earlier studies have also argued that interactions are size‐based and most common between predators with intermediate body‐size ratios (Donadio & Buskirk, 2006; Palomares & Caro, 1999; Prugh & Sivy, 2020). However, the widespread predation and killing of our smallest predators, weasels, and stoats (victim to eight and five predator taxa, respectively), could suggest that previous research may have overlooked interactions with small predators owing to the constrains imposed by their small sizes.

### 4.2 Is predator suppression the norm?

We only found evidence that three of the 12 predator taxa included in this review were under some degree of top‐down suppression through lethal predator interactions, namely the Eurasian lynx suppressing red fox, red fox suppressing pine marten, and northern goshawk suppressing kestrel. Diet studies provide the only type of data found consistently for these three pairs, and only red foxes in the diet of lynx occurs at a frequency of occurrence above 2% (Table 2), stressing the impossibility to infer demographic impacts on intraguild prey from diet data alone. The proportion of predator interactions leading to suppression was only 6.8%, which may suggest that population‐level effects are rare relative to the prevalence of predator interactions. This could be due to several factors. Our focus on suppression caused by lethal interactions alone may have underestimated the number of taxa under some form suppression. Certainly, despite representing few interactions, suppressed predators account for 25% of this review's taxa, and additional instances would change this figure considerably. Furthermore, combining multiple types of data may have revealed a larger number of interactions than previous studies based on killings only. For instance, Palomares and Caro (1999) detected 1.6 interactions per species (97 interactions between 60 unique species), while we uncovered 3.7 interactions per taxa. Lastly, most lethal interactions (38 of 44) remain unevaluated for population‐level effects, highlighting the uncertainty surrounding their prevalence. Consequently, despite the potential of population‐level effects, they should not be presumed even of known predator interactions without evidence to support them.

### 4.3 Requirements for suppression

In addition to three interactions leading to suppression, another three had evidence allowing us to conclude, provisionally, that no population suppression occurs. These are golden eagles versus red foxes and pine martens, and northern goshawks versus tawny owls. The latter offers insights on why some interactions may cause demographic suppression of the victim while others do not. Unlike kestrels, the population of tawny owls did not decline despite similar predation rates by recovering goshawks because predation was disproportionately directed toward senescent adult females and juveniles, two stages with low reproductive output (Hoy et al., 2015; Petty et al., 2003). This contrast between the two species is in line with the expectation that survival rates of both senescent and juveniles have low elasticities to the population's growth rate compared with breeding adults in long‐lived species (Hoy et al., 2015). We suggest that such a demographic perspective offers a useful framework within which to evaluate the expected population impact of predator interactions. For instance, golden eagles did not suppress red foxes in Finland despite the ubiquitous, albeit low frequency, occurrence of fox in the eagle's diet (Lyly et al., 2015; Table 2). Contrastingly, golden eagles strongly suppressed the smaller island fox (*Urocyon littoralis*) population of the Channel Islands, California, (Roemer et al., 2002). The difference in size between the two species of foxes offers a plausible explanation. Owing to their smaller size, both island fox adults (with high survival elasticity) and young are vulnerable to predation, whereas it is mostly young red foxes (with low survival elasticity) that fall prey to golden eagles (Tjernberg, 1981). Eurasian lynx killing adult red foxes and foxes killing adult pine marten are two further instances of population suppression where an influential demographic stage is the victim. It is striking, however, that demographic suppression arises for these pairs given the relatively low mortalities inflicted by lynx on fox (4%–14%), and fox on marten (13%), compared with the high harvesting‐related mortality of an American marten (*Martes americana*) population of 37.9%, which was unrelated to population density (Fryxell et al., 1999; Helldin et al., 2006; Lindström et al., 1995). This may suggest that lynx‐ or fox‐induced mortality is fully additive to other causes of mortality. Therefore, we posit that population suppression of vertebrate predators through direct predator interactions (i.e., intraguild predation and killing) is contingent on impacting a stage of the victim population with high survival elasticity.

According to our elasticity hypothesis, we would not expect arboreal pine martens, or any terrestrial predator, to impose population‐level suppression on avian predators as they predate mostly eggs and chicks with low survival elasticities (Table S2). Indeed, adult owls using cavity nests with entrances large enough to allow martens through, explore disturbances (e.g., scratching sounds on the tree) more often than smaller owls (Sonerud, 1985). This is an instance of a predator‐avoidance strategy that canalizes adult survival, the trait with greatest impact on the population growth rate, against temporal variability (Gaillard & Yoccoz, 2003). Under this hypothesis, pine martens would be potential candidates for suppression by golden eagles as, unlike red foxes, adult martens are on the eagle's prey‐size spectrum (Högström & Wiss, 1992). Nonetheless, a lower overlap in their diets, the low densities of golden eagles and more importantly, the refuge that forest habitats offer to pine martens would reduce the frequency of interactions between the two and possibly explain why pine martens are not significantly suppressed by golden eagles (Lyly et al., 2015). Consequently, we can point out two requirements for predator interactions to cause population suppression: (i) the ability to impact a demographic component of their victim population that has high elasticity and (ii) a motivation for these interactions, such as competition for a resource (e.g., ungulate carcasses), or where the victim predator itself is a potential prey, that leads to a sufficiently high frequency of interaction. Although the elasticity hypothesis presented here is entrenched in demography, it has not been used in the context of predator interactions. Further validation of this hypothesis is required and could be achieved by: (i) using the fragmented distribution of large predators’ or the natural experiments offered by their recovery or extirpation in areas of their range, to facilitate detection of demographic impacts and (ii) monitoring multiple predator victims that have either similar age class elasticities, but different classes affected by the larger predator, or vice versa. Long‐term bird‐ringing programs and traditional diet studies were able to do this with northern goshawks (Hoy et al., 2017) but are not transferable to mammal predators. Instead, GPS or VHF‐based tracking would allow monitoring different causes of mortality of different demographic groups and species.

### 4.4 Suppression versus displacement

Scarce and conflicting evidence on the extent and mechanisms of population suppression precludes evaluation of the interaction between eagle owls and goshawks and between northern goshawks and common buzzards. There was a single study for the former pair with an apparent release of a buzzard population following a goshawk decline, but this could also be attributed to changing habitat and environmental conditions (Gryz & Krauze‐Gryz, 2019). Additionally, this process was not matched in Kielder Forest, northern England, where buzzards increased as goshawks recolonized the area (Martin Davidson unpublished). The evidence of goshawk suppression by eagle owls stems from three papers, two of which reached contrasting conclusions despite using largely the same data (Chakarov & Krüger, 2010; Mueller et al., 2016). While eagle owl and goshawk impacts on the breeding success of their victims are well documented, according to our elasticity hypothesis, this alone should not translate into a population impact. A limitation of studies with raptors is that evidence of lethal interactions between raptors is largely restricted to dietary studies during the bird's breeding season, and although goshawks and buzzards occur in the diet of eagle owls and goshawks, respectively, it is in low proportions and mostly involving juveniles (e.g., Kostrzewa, 1991). The nature of the evidence implies that the killing of adults without consumption would remain mostly undetected. Therefore, we cannot presently assess the importance of such interactions. Studying the diet of large raptors outside the breeding season and beyond what is brought back to the nest, particularly ascertaining whether they engage in killings without consumption, would contribute greatly to our understanding of raptor interactions. High‐resolution GPS tracking could allow the identification of kill sites that could be validated in the field (e.g., Sivy et al., 2017) and of roosting sites where fecal matter could be collected for genetic‐based diet analysis (e.g., Hopkins, 2019).

The observed patterns of population depression in the eagle owl‐goshawk case study above could alternatively be the result of individual displacement combined with small study sites relative to the bird's mobility. Krüger (2004) noted that lower breeding output of buzzards near goshawk nests was caused by nest abandonments, and dispersing birds resettling beyond the study area would create the impression of a local suppression. The contrasting results of Chakarov and Krüger (2010) and Mueller et al. (2016) may thus be reconciled by considering different divisions of the same study area where the former, by dividing it into three subareas, detected a displacement of goshawks caused by recolonizing eagle owls, that the latter could not. This is characterized in detail by Sergio et al. (2007) where due to risk‐avoidance strategies (i.e., indirect or non‐lethal interactions) the proportion of the landscape available to tawny owls decreased with increasing eagle owl densities. This reduced available nesting sites and increased intraspecies competition, ultimately leading to strong negative correlations between the breeding densities of both owl populations. A growing body of evidence addresses the effects of indirect, non‐lethal, sub‐lethal, or non‐consumptive predator interactions (e.g., Clinchy et al., 2013; Lima, 1998; McCauley et al., 2011). However, the evidence that these alone can depress wild populations is scarce, particularly in terrestrial vertebrate communities (Sheriff et al., 2020). Future work should strive to discern the relative contribution toward population suppression of direct and indirect mechanisms of interaction. For instance, GPS tracking both killer and victim predators throughout a range of killer densities (i.e., risk levels). This would allow not only quantifying direct mortality, but also the magnitude of avoidance/displacement and its potential population‐level effects through the reduction in available landscape and resources.

### 4.5 Other candidates for suppression

Predation of small mustelids by raptors, red foxes, and pine martens alone accounts for 14 out of 35 interactions for which we lack evidence for or against demographic impacts (not including the three interactions among raptors discussed in the section above). Hitherto, inherent difficulties of monitoring small mustelid populations have prevented the detection of their suppression or lack thereof (Lambin, 2017). Given their life histories, with shorter lifespans and higher fecundity, we do not expect any one predator to exert suppression on small mustelids through predation (Macdonald & Newman, 2017). However, small mustelids are exposed to predation by up to eight largely sympatric predator taxa. Therefore, it would be conceivable that the combined pressure of all predators translates into significant levels of top‐down suppression of small mustelid populations. A similar case can be made for corvids as they are subject to considerable predation by all four large raptors considered in this review (4 interactions). With average frequencies of occurrences ranging between 2.3% and 17.1%, and holding the three largest occurrences documented in this review (36.2%, 21.7%, 18.2%; Table 2), even if a sizeable proportion of predation focused on nonbreeders, corvids could be under high pressure where multiple large raptors are sympatric. This could also be true, though to a lesser degree, for smaller diurnal raptors and owls (4 interactions each). Unlike in the previous case, however, small raptors make smaller contributions to the diet of large raptors (range: 0.2%–1.8% FO). Additionally, we have seen that goshawks are both killer and victim in interactions potentially leading to suppression/displacement with common buzzards and eagle owls, respectively. Hence, it is difficult to draw inferences on the potential cumulative effects of large raptor predation on smaller raptors. As for the remaining 12 interactions between raptors and nonmustelid mammals (both directions), Eurasian lynx and pine marten, mammals and corvids, and between golden eagles, eagle owls and other large raptors, we do not consider them as plausible candidates to cause population suppression for reasons already discussed, including the lack of impact on age classes with high survival elasticity, and differing niches reducing the frequency of interaction. To conclude, the above evidence highlights the need to move beyond pairs of interacting species (Jachowski et al., 2020; Prugh & Sivy, 2020). Indeed, with eight out of 12 taxa falling victim to three or more predators, considering synergies between multiple interactions could reveal lethal predator interactions as a stronger driver of predator guild's structure than it transpires from this review.

### 4.6 Carcasses as an interaction hub for mammals

All mammalian predators studied here except weasels engaged in interspecific killings (i.e., killing without consuming the victim) of other grouse‐predator mammals (Table S2). After finding at least five unconsumed small mustelids in red fox dens, Latham (1952) proposed that they may be distasteful to mammals. We found similar evidence with large proportions of uneaten weasels (e.g., 51 out of 54 in Belarus; Sidorovich, 2011). Thus, a possible explanation could be that they are killed but not eaten when predators of grassland small mammals blindly attempt capturing prey they hear but do not see (Lambin, 2017). Otherwise, killing but not consuming readily available food (i.e., the killed predator) may be the result of extreme interference competition over carcasses. For instance, in North America, ​a cougar (*Felis concolor*) killed but not consumed a coyote near a prey carcass but killed and ate four when no carcass was nearby (Boyd & O’Gara, 1985 as cited by Palomares & Caro, 1999). Similarly, female Eurasian lynx and their cubs, known to consume larger proportions of their kills than males by returning more often to them, also tend to kill red foxes at higher rates than males (Molinari‐Jobin et al., 2002; Sunde et al., 2000). Foxes that are killed to protect a kill would be of little trophic value compared with the carcass itself. If exploiting another predator's kill incurs a high risk of mortality, instead of providing a valuable resource, carcasses may operate as ecological traps and be instrumental in suppression arising between some mammalian predators, as proposed by the fatal attraction hypothesis (Prugh & Sivy, 2020; Sivy et al., 2017). This may explain the seemingly contradicting findings of Helldin et al. (2006) and Helldin and Danielsson (2007), where following the recolonization of lynx to southern Sweden, the proportion of ungulates in the diet of red foxes increased by 21.2%, concomitant with an annual decline of 10.5% in the fox population. It also offers an explanation for the observed positive association between the two species at a small scale, reflecting foxes being attracted to lynx kills (Wikenros et al., 2017) but a negative association over a much larger area as foxes are removed by lynx (Pasanen‐Mortensen et al., 2017). The role of carcasses in modulating the strength of interactions between predators is rapidly gaining attention as a powerful incentive for interspecific killing of mammals (e.g., Prugh & Sivy, 2020).

### 4.7 Drivers of predator interactions

Predator interactions have been hypothesized to be driven by the removal of competitors and food limitation (see Lourenço et al., 2018). The data compiled here seemingly indicates that different groups of predators may have different underlying motivations. The abundant evidence of interspecific killing without consumption between mammals is consistent with the removal of competitors. Sunde et al. (1999), for instance, observed that the proportion of completely unconsumed foxes was 37% (7 of 19) compared to the much smaller proportion of uneaten hares (*Lepus* spp.), 0% (0 of 15), and roe deer (*Capreolus capreolus*), 2% (1 of 44). Instead, we have seen how large raptors, particularly northern goshawks but also golden eagles and common buzzards, use other predators (e.g., corvids, smaller raptors) as alternative prey when they are food limited (Clouet et al., 2017; Hoy et al., 2017; Rooney & Montgomery, 2013; Tornberg et al., 2009; Watson, 1998), which is also consistent with the alternative prey hypothesis (Angelstam et al., 1984; Breisjøberget et al., 2018). Similar evidence was found in the Iberian Peninsula, where the occurrence of avian predators in the diet of eagle owls was negatively correlated to their main prey's abundance, rabbits (*Oryctolagus cuniculus*; Lourenço et al., 2018; Serrano, 2000). Thus, food limitation provides a “motivation” for a high frequency of intraguild predation and, bearing in mind the methodological limitations of raptor diet studies highlighted earlier, it seems that raptors simply view other (smaller) avian predators as potential prey to fall back on. Interactions between mammals are seemingly more complex, as they engage both in interspecific killing and intraguild predation.

### 4.8 Predator interactions and conservation

One motivation of this review was to evaluate the importance of predator interactions when planning the conservation of European forest grouse. Forest grouse are ground‐nesting birds with single, yearly broods vulnerable to predation (Roos et al., 2018). Consequently, predator control is a common management intervention (e.g., Summers et al., 2004). The reintroduction of top predators that could suppress other “problematic” predators has been proposed too (e.g., Moreno‐Opo et al., 2015). The evidence gathered here highlights both the prevalence of predator interactions and the large uncertainties that remain around them. Failing to consider predator interactions can critically affect the success of conservation actions. For instance, in two carnivore reintroduction programs of swift fox (*Vulpes velox*) and black‐footed ferret (*Mustela nigripes*) in North America, predation accounted for 91% and 100% of known causes of mortality, respectively (Carbyn et al., 1994; Clark, 1994). Predator control may benefit prey populations, including forest grouse (Kämmerle & Storch, 2019; Marcstrom et al., 1988; Smith et al., 2010). However, benefits are often short‐lived (Lieury et al., 2015; Moreno‐Opo et al., 2015; Newsome et al., 2014). Predator interactions (or lack thereof) may be a contributing factor where the loss of top‐down pressure releases nontargeted predators, which may, in turn, offset the accomplishments of predator control (Crooks & Soulé, 1999; Lennox et al., 2018; Ripple et al., 2013). Equally, there is presently no compelling evidence that top predators protect European forest grouse through the suppression or displacement of other predators (Lyly et al., 2016; Mönkkönen et al., 2007; Tornberg et al., 2016). Forest‐grouse conservation in Europe is a multifacetted problem that no silver bullet will resolve. Science, management, and policy should work hands in hands to identify the sources of uncertainty of different management practices and implement reasoned conservation strategies for the sustainable protection of vulnerable prey species.

## CONFLICTS OF INTEREST

There are no conflicts of interest to report by the authors.

## AUTHOR CONTRIBUTION

**Cristian N. Waggershauser:** Conceptualization (equal); Data curation (lead); Formal analysis (lead); Investigation (equal); Methodology (lead); Software (lead); Writing‐review & editing (lead). **Lise Ruffino:** Conceptualization (equal); Data curation (supporting); Investigation (equal); Methodology (supporting); Writing‐original draft (lead); Writing‐review & editing (supporting). **Kenny Kortland:** Conceptualization (equal); Funding acquisition (equal); Writing‐review & editing (supporting). **Xavier Lambin:** Conceptualization (equal); Funding acquisition (equal); Methodology (supporting); Project administration (lead); Supervision (lead); Writing‐review & editing (supporting).

## Supporting information

Table S1

Table S2

Table S3

Appendix S1

## Data Availability

All data used in this review is available from the Dryad Data Repository: https://doi.org/10.5061/dryad.79cnp5hvb
