# Supplementary material for: Lethal interactions among forest‐grouse predators are numerous, motivated by hunger and carcasses, and their impacts determined by the demographic value of the victims
Source: Ecol Evol. 2021 May 2;11(12):7164–86. doi: 10.1002/ece3.7574 (PMC8216895; doi:10.1002/ece3.7574)

**ELECTRONIC SUPPLEMENTARY MATERIAL**

**S4. References of reviewed studies.**

Austruy, J.-C., & Cugnasse, J.-M. (1981). L’aigle royal, Aquila chrysaetos, dans le massif central. *Le Grand Duc*, *20*, 3–9.

Baker, P., Furlong, M., Southern, S., & Harris, S. (2006). The potential impact of red fox Vulpes vulpes predation in agricultural landscapes in lowland Britain. *Wildlife Biology*, *12*(1), 39–50. https://doi.org/10.2981/0909-6396(2006)12[39:TPIORF]2.0.CO;2

Bezzel, E., Obst, J., & Wickl, K. H. (1976). On the diet of the eagle owl (Bubo bubo) [In German]. *Journal of Ornithology*, *117*(2), 210–238. https://doi.org/10.1007/BF01643587

Birkeland, K. H., & Myrberget, S. (1980). The diet of the lynx Lynx lynx in Norway. *Fauna Norvegica*, *1*, 24–28.

Brzeziński, M., Rodak, Ł., & Zalewski, A. (2014). “Reversed” intraguild predation: Red fox cubs killed by pine marten. *Acta Theriologica*, *59*(3), 473–477. https://doi.org/10.1007/s13364-014-0179-8

Busche, G., Raddatz, H. J., & Kostrzewa, A. (2004). Nistplatz-Konkurrenz und Prädation zwischen Uhu (Bubo bubo) und Habicht (Accipiter gentilis): erste Ergebnisse aus Norddeutschland [In German]. *Vogelwarte*, *42*, 169–177. https://www.zobodat.at/pdf/Vogelwarte_42_2003_0169-0177.pdf

Byholm, P., & Nikula, A. (2007). Nesting failure in Finnish Northern Goshawks Accipiter gentilis: Incidence and cause. *Ibis*, *149*(3), 597–604. https://doi.org/10.1111/j.1474-919X.2007.00687.x

Capt, S. F., Bernhary, F., Breitenmoser, U., Breitenmoser-Würsten, C., Haller, H., Liberek, M., Vandel, J. M., & Herrenschmidt, V. (1993). Predation du lynx (Lynx lynx) sur les ongules sauvages et domestiques. In P. Migot & P. Stahl (Eds.), *Colloque prédation et gestion des prédateurs* (pp. 85–92). Union National des Federations de Chasse.

Chakarov, N., & Krüger, O. (2010). Mesopredator Release by an Emergent Superpredator: A Natural Experiment of Predation in a Three Level Guild. *PLoS ONE*, *5*(12), e15229. https://doi.org/10.1371/journal.pone.0015229

Clouet, M. (1981). L’aigle royal Aquila chrysaetos dans les Pyrénées francaises. Résultats de 5 ans d’observations. [In French]. *L’Oiseau*, *51*, 89–100.

Clouet, M., Gerard, J. F., Goar, J. L., Goulard, M., González, L., Rebours, I., & Faure, C. (2017). Diet and Breeding Performance of the Golden Eagle Aquila Chrysaetos at the Eastern and Western Extremities of the Pyrenees: An Example of Intra-Population Variability. *Ardeola*, *64*(2), 347–361. https://doi.org/10.13157/arla.64.2.2017.ra4

Cugnasse, J.-M. (1983). Contribution à l’étude du Hibou grand-duc Bubo bubo dans le sud du Massif Central. *Nos Oiseaux*, *37*(3), 117–128.

Dell’Arte, G. L., Laaksonen, T., Norrdahl, K., & Korpimäki, E. (2007). Variation in the diet composition of a generalist predator, the red fox, in relation to season and density of main prey. *Acta Oecologica*, *31*(3), 276–281. https://doi.org/https://doi.org/10.1016/j.actao.2006.12.007

Dihle, I. (2015). *Prey selection and prey handling in two raptors during the breeding season as revealed by the use of video monitoring* [Norwegian University of Life Sciences]. http://hdl.handle.net/11250/295797

Dunker, H. (1988). Winter studies on the lynx (Lynx lynx) in southeastern Norway from 1960-1982. *Meddelelser Fra Norsk Vilforskning*, *3*, 1–56.

Elmhagen, B., Ludwig, G., Rushton, S. P., Helle, P., & Lindén, H. (2010). Top predators, mesopredators and their prey: interference ecosystems along bioclimatic productivity gradients. *Journal of Animal Ecology*, *79*(4), 785–794. https://doi.org/10.1111/j.1365-2656.2010.01678.x

Elmhagen, B., & Rushton, S. P. (2007). Trophic control of mesopredators in terrestrial ecosystems: Top-down or bottom-up? *Ecology Letters*, *10*(3), 197–206. https://doi.org/10.1111/j.1461-0248.2006.01010.x

Emmett, R. E., Mikkola, H., Mummery, L., & Westerhoff, G. (1972). Prey found in Eagle Owls’ nest in central Sweden. *British Birds*, *65*, 482–483. https://www.researchgate.net/publication/280575991_Emmett_RE_Mikkola_H_Mummery_L_Westerhoff_G_1972_Prey_found_in_Eagle_Owl%27s_nest_in_central_Sweden_British_Birds_65_482-483

Erfurt, J., & Stubben, M. (1987). Geöllanalysen zur Untersuchung der Ernährungsbiologie von Eulen. *Populationsökologie Greifvogel Eulenarten [In German]*, *1*, 429–451.

Frey, H. (1973). Zur Okologie niederosterreichischer Uhupopulationen [In German]. *Egretta*, *16*, 1–66.

Frey, H., & Walter, W. (1977). Brutvorkommen und Nahrungsokologie des Uhus (Bubo bubo) im Burgenland [In German]. *Egretta*, *20*, 26–35.

Frey, H., & Walter, W. (1986). Zur Ernährung des Uhus, Bubo bubo (Linnaeus 1758), Aves, an einem alpinen Brutplatz in den Hohen Tauern (Salzburg, Ósterreich)[In German]. *Annalen Des Naturhistorischen Museums in Wien*, *88/89*, 91–99.

Gaspar, G., Misiewicz, A., Armatys, P., Loch, J., Czarnota, P., & Wierzbowski, J. (2018). Diet composition of carnivore species in the Gorce National Park. *Sylwan*, *162*(04). https://doi.org/10.26202/sylwan.2017097

Glöersen, G. (1996). *Rapport fran lo- och varginventeringen [In Swedish]*.

Glue, D., & Hammond, G. (1974). Feeding ecology of the Longeared Owl in Britain and Ireland. *Bird Study*, *21*, 200–210.

Glutz von Blotzheim, U. N., Bauer, K. N., & Bezzel, E. (1971). *Handbuch der Vögel Mitteleuropas. Band 4. Falconiformes. [In German]*. Akademische Verlagsgescellschaft.

Goszczyński, J. (1991). The food habit of Buzzards and Goshawks during the nesting period. In S. Csányi & J. Ernhaft (Eds.), *XXth Congress International of the Union of Game Biologists* (pp. 377–390). University of Agricultural Sciences.

Goszczyński, J., & Pilatowski, T. (1986). Diet of Common Buzzards (Buteo buteo L.) and Goshawks (Accipiter gentilis L.) in the nesting period. *Ekologia Polska*, *34*, 655–667.

Goszczyński, J. (1976). Composition of the food of martens. *Acta Theriologica*, *21*, 527–534. https://doi.org/10.4098/at.arch.76-46

Goszczyński, J. (1974). Studies on the food of foxes. *Acta Theriologica*, *19*, 1–18. https://doi.org/10.4098/at.arch.74-1

Graham, I. M., Redpath, S. M., & Thirgood, S. J. (1995). The diet and breeding density of common buzzards buteo buteo in relation to indices of prey abundance. *Bird Study*, *42*(2), 165–173. https://doi.org/10.1080/00063659509477162

Grönlund, S., & Mikkola, H. (1969). Suopöllön ekologiasta Lapuan Alajoella v. [In Finnish]. *Suomenselän Linnut*, *4*, 68–76.

Gryz, J., & Krauze-Gryz, D. (2019). The common buzzard buteo buteo population in a changing environment, central Poland as a case study. *Diversity*, *11*(3), 35. https://doi.org/10.3390/D11030035

Gryz, J., & Krauze-Gryz, D. (2014). The influence of raptors (Falconiformes) and ravens (Corvus corax) on populations of game animals. In *Annals of Warsaw University of Life Sciences - SGGW. Forestry and Wood Technology* (Vol. 86). -.

Hakkarainen, H., & Korpimaki, E. (1996). Competitive and Predatory Interactions among Raptors: An Observational and Experimental Study. *Ecology*, *77*(4), 1134–1142. https://doi.org/10.2307/2265582

Hakkarainen, H., Mykrä, S., Kurki, S., Tornberg, R., & Jungell, S. (2004). Competitive interactions among raptors in boreal forests. *Oecologia*, *141*(3), 420–424. https://doi.org/10.1007/s00442-004-1656-6

Halgund, B. (1966). De stora rovdjurens vintervanor [In Swedish]. *Viltrevy*, *4*, 1–311.

Halliwell, E. (1997). *The Ecology of Red Squirrels in Scotland in relation to Pine Marten Predation*. University of Aberdeen.

Hell, P. (1978). Die situation des Karpatenluchses (Lynx lynx) in der Tschechoslowakei [In German]. In U. Wotschikowsky (Ed.), *Der luchs. Erhaltun und Wiedereinbürgerung in Europa*. Bernhard.

Helldin, J. O. (2000). Seasonal diet of pine marten Martes martes in southern boreal Sweden. *Acta Theriologica*, *45*(3), 409–420. https://doi.org/10.4098/AT.arch.00-40

Helldin, J. O. (1999). Diet, body condition, and reproduction of Eurasian pine martens Martes martes during cycles in microtine density. *Ecography*, *22*(3), 324–336. https://doi.org/10.1111/j.1600-0587.1999.tb00508.x

Helldin, J. O., & Danielsson, A. V. (2007). Changes in red fox Vulpes vulpes diet due to colonisation by lynx Lynx lynx. *Wildlife Biology*, *13*(4), 475–480. https://doi.org/10.2981/0909-6396(2007)13[475:CIRFVV]2.0.CO;2

Helldin, J. O., Liberg, O., & Glöersen, G. (2006). Lynx (Lynx lynx) killing red foxes (Vulpes vulpes) in boreal Sweden - Frequency and population effects. *Journal of Zoology*, *270*(4), 657–663. https://doi.org/10.1111/j.1469-7998.2006.00172.x

Högström, S., & Wiss, L.-E. (1992). Diet of the Golden Eagle Aquila chrysaetos ( L .) in Gotland, Sweden during the breeding season. *Ornis Fennica*, *69*(1), 39–44.

Hoy, S. R., Petty, S. J., Millon, A., Whitfield, D. P., Marquiss, M., Anderson, D. I. K. K., Davison, M., & Lambin, X. (2017). Density-dependent increase in superpredation linked to food limitation in a recovering population of northern goshawks Accipiter gentilis. *Journal of Avian Biology*, *48*, 1205–1215. https://doi.org/10.1111/jav.01387

Hoy, S. R., Petty, S. J., Millon, A., Whitfield, D. P., Marquiss, M., Davison, M., & Lambin, X. (2015). Age and sex-selective predation moderate the overall impact of predators. *Journal of Animal Ecology*, *84*(3), 692–701. https://doi.org/10.1111/1365-2656.12310

Huboux, R. (1987). Contribution à une meilleure connaissance du régime alimentaire de l’Aigle Royal en periode de reproduction pour les Alpes de Sud at la Provence [In French]. In S. Michel (Ed.), *L’Aigle Royal en Europe.* (pp. 118–123). Actes du Premier Colloque International.

Huhtala, K. (1976). Kanahaukan ravinnosta [In Finnish]. *Suomen Luonto*, *35*, 304–306.

Ims, R. A. J. (1982). Førekomst, habitat og byttevalg hos haukugle i et smågnagerår i Ost-Finmark 1978 [In Norwegian]. *Fauna*, *35*, 133–139.

Iselin, H., & Hämmerle, E. (1960). Beobachtungen am Adlerhorst: Der Nahrungsbedarf zweier Jungadler [In German: Observations at the eagle’s nest: the food requirements of two young eagles]. *Ornitologischer Beobachter*, *57*, 129–132. https://www.ala-schweiz.ch/index.php/ornithologischer-beobachter/artikel-suche?indexid=1602

Ivanosvsky, V. V. (1990). [Birds of prey and ornithological monitoring] In Russian. *Study of Bird Migration*, *22*, 92–101.

Jędrzejewska, B., & Jędrzejeweski, W. (2001). *[Ecology of predators of Bialowiea Primeval Forest] In Polish*. Polish Scientific Publishers.

Jędrzejewski, W., Szymura, A., & Jędrzejewska, B. (1994). Reproduction and food of the buzzard buteo buteo in relation to the abundance of rodents and birds in bialowieza national park, poland. *Ethology Ecology and Evolution*, *6*(2), 179–190. https://doi.org/10.1080/08927014.1994.9522993

Jedrzejewski, W., Zalewski, A., & Jedrzejewska, B. (1993). Foraging by pine marten Martes martes in relation to food resources in Bialowieza National Park, Poland. *Acta Theriologica1*, *38*, 405–426.

Jędrzejewski, W., & Jędrzejewska, B. (1992). Foraging and diet of the red fox Vulpes vulpes in relation to variable food resources in Biatowieza National Park, Poland. *Ecography*, *15*(2), 212–220. https://doi.org/10.1111/j.1600-0587.1992.tb00027.x

Jobin, A., Molinari, P., & Breitenmoser, U. (2000). Prey spectrum, prey preference and consumption rates of Eurasian lynx in the Swiss Jura Mountains. *Acta Theriologica*, *45*(2), 243–252. https://doi.org/10.4098/AT.arch.00-26

Johansen, H. M., Selås, V., Fagerland, K., Johnsen, J. T., Sveen, B. A., Tapia, L., & Steen, R. (2007). Goshawk diet during the nestling period in farmland and forest-dominated areas in southern Norway. *Ornis Fennica*, *84*(4), 181–188.

Johnsen, T. V., Systad, G. H., Jacobsen, K. O., Nygård, T., & Bustnes, J. O. (2007). The occurrence of reindeer calves in the diet of nesting Golden Eagles in Finnmark, northern Norway. *Ornis Fennica*, *84*(3), 112–118.

Kidawa, D., & Kowalczyk, R. (2011). The effects of sex, age, season and habitat on diet of the red fox Vulpes vulpes in northeastern Poland. *Acta Theriologica*, *56*(3), 209–218. https://doi.org/10.1007/s13364-011-0031-3

Korpimäki, E. (1988). Diet of breeding Tengmalm’s owls Aegolius funereus: Long-term changes and year-to-year variation under cyclic food conditions. *Ornis Fennica*, *65*(1), 21–30.

Korpimaki, E. (1986). Niche relationships and life-history tactics of three sympatric Strix owl species in Finland. *Ornis Scandinavica*, *17*(2), 126–132. https://doi.org/10.2307/3676861

Korpimäki, E., Huhtala, K., Sulkava, S., & Korpimaki, E. (1990). Does the Year-to-Year Variation in the Diet of Eagle and Ural Owls Support the Alternative Prey Hypothesis? *Oikos*, *58*(1), 47. https://doi.org/10.2307/3565359

Korpimäki, E., & Norrdahl, K. (1989). Avian Predation on Mustelids in Europe 1: Occurrence and Effects on Body Size Variation and Life Traits. *Oikos*, *55*(2), 205–215. https://doi.org/10.2307/3565424

Korpimäki, E., & Sulkava, S. (1987). Diet and breeding performance of Ural Owls Strix uralensis under fluctuating food conditions. *Ornis Fennica*, *64*, 57–66.

Kostrzewa, A. (1991). Interspecific interference competition in three european raptor species. *Ethology Ecology and Evolution*, *3*(2), 127–143. https://doi.org/10.1080/08927014.1991.9525379

Krauze, D., Gryz, J., & Goszczyński, J. (2005). Food composition of the goshawk (Accipiter gentilis L. 1758) during the nesting season in the Rogów Forest (central Poland). *Folia Forestalia Polonica, Series A*, *47*(47), 45–53.

Krüger, O. (2004). The importance of competition, food, habitat, weather and phenotype for the reproduction of Buzzard Buteo buteo. *Bird Study*, *51*(2), 125–132. https://doi.org/10.1080/00063650409461344

Krüger, O. (2002). Interactions between common buzzard Buteo buteo and goshawk Accipiter gentilis: trade-offs revealed by a field experiment. *Oikos*, *96*(3), 441–452. https://doi.org/10.1034/j.1600-0706.2002.960306.x

Kurki, S., Nikula, A., Helle, P., Lindén, H., & Linden, H. (1998). Abundances of red fox and pine marten in relation to the composition of boreal forest landscapes. *Journal of Animal Ecology*, *67*(6), 874–886. https://doi.org/10.1046/j.1365-2656.1998.6760874.x

Lanszki, J. (2005). Diet composition of red fox during rearing in a moor: A case study. *Folia Zoologica*, *54*(1–2), 213–216.

Lanszki, J., Zalewski, A., & Horváth, G. (2007). Comparison of red fox Vulpes vulpes and pine marten Martes martes food habits in a deciduous forest in Hungary. *Wildlife Biology*, *13*(3), 258–271. https://doi.org/10.2981/0909-6396(2007)13[258:CORFVV]2.0.CO;2

Laursen, J. (1999). Fødevalg hos Stor Hornugle Bubo bubo i Danmark [In Danish]. *Danks Ornitologisk Forenings Tidsskrift*, *93*, 141–144.

Liberg, O. (1997). *Lodjuret*. Svenska Jägareförbundet.

Lindström, E. R., Brainerd, S. M., Helldin, J. O., & Overskaug, K. (1995). Pine Marten Red Fox Interactions - a Case of Intraguild Predation. *Annales Zoologici Fennici*, *32*(1), 123–130. //a1995rb12100016

Linnell, J. D. C., Odden, J., Pedersen, V., & Andersen, R. (1998). Records of intra-guild predation by Eurasian Lynx, Lynx lynx. *Canadian Field-Naturalist*, *112*(4), 707–708.

Lockie, J. D., & Stephen, D. (1959). Eagles, Lambs and Land Management on Lewis. *The Journal of Animal Ecology*, *28*(1), 43. https://doi.org/10.2307/2012

Lohmus, A. (1993). Kanakulli (Accipiter gentilis) Toitumisest eestis Aastatel 1987-92 [In Estonian]. *Hirundo*, *2*(13), 3–14.

Lyly, M. S., Villers, A., Koivisto, E., Helle, P., Ollila, T., & Korpimäki, E. (2015). Avian top predator and the landscape of fear: Responses of mammalian mesopredators to risk imposed by the golden eagle. *Ecology and Evolution*, *5*(2), 503–514. https://doi.org/10.1002/ece3.1370

Lynch, Á. B., & McCann, Y. (2007). The diet of the pine marten (Martes martes) in Killarney National Park. In *Biology and Environment* (Vol. 107, Issue 2). https://doi.org/10.3318/BIOE.2007.107.2.67

Marchesi, L., Sergio, F., & Pedrini, P. (2002). Costs and benefits of breeding in human-altered landscapes for the Eagle Owl Bubo bubo. *Ibis*, *144*(4), E164–E177. https://doi.org/10.1046/j.1474-919x.2002.t01-2-00094_2.x

Marchesi, P., & Mermod, C. (1989). Diet of the pine marten (Martes martes) in Switzerland. *Revuew Suisse de Zoologie*, *96*(1), 127–146.

Marquiss, M., & Newton, I. (1982). The Goshawk in Britain. *Brittish Birds*, *75*, 243–260.

Marquiss, M., Ratcliffe, D. A., & Roxburgh, R. (1985). The numbers, breeding success and diet of golden eagles in southern Scotland in relation to changes in land use. *Biological Conservation*, *34*(2), 121–140. https://doi.org/10.1016/0006-3207(85)90104-1

Martin, Y., & Riols, C. (2017). Régime alimentaire du Grand-duc d’Europe (Bubo bubo) dans le Puy-de-Dôme et sur le prolongement du bassin de la Sioule dans le département de l’Allier (Auvergne). [In French]. *Le Grand-Duc*, *85*, 9–30.

Mathieu, R., & Choisy, J. P. (1982). L’Aigle Royal Aquila chrysaetos dans les Alpes Meridionales francaises de 1964 a 1980. Essai sur la distribution, les effectifs, le régime alimentaire et la reproduction. [In French]. *Bièvre*, *4*, 1–32.

Mebs, T. (1964). Zur Biologie und Populationsdynamik des Mäusebussards(Buteo buteo) (Unter besonderer Berücksichtigung der Abhängigkeit vom Massenwechsel der Feldmaus Microtus arvalis) [In German]. *Journal Für Ornithologie*, *105*(3), 247–306. https://doi.org/10.1007/BF01672243

Mikkola, H. (1983). *Owls of Europe*. T. & A. D. Poyser.

Mueller, A., Chakarov, N., Heseker, H., & Krüger, O. (2016). Intraguild predation leads to cascading effects on habitat choice, behaviour and reproductive performance. *Journal of Animal Ecology*, *85*(3), 774–784. https://doi.org/10.1111/1365-2656.12493

Nielsen, J. T. (2003). DuehøgensAccipiter gentilisbyttevalg uden for yngletiden [In Danish]. *Danks Ornitologisk Forenings Tidsskrift*, *97*, 193–198.

Nielsen, J. T., & Drachmann, J. (1999). Prey selection of Goshawks Accipiter gentilis during the breeding season in Vendsyssel. *Danks Ornitologisk Forenings Tidsskrift*, *93*, 85–90.

Nygård, M. (2015). *Diet and prey handling at a nest of the golden eagle (Aquila chrysaetos) in Oppland County, Norway* [Norwegian University of Life Sciences]. http://hdl.handle.net/11250/2383515

Nyström, J., Ekenstedt, J., Angerbjörn, A., Thulin, L., Hellström, P., & Dalén, L. (2006). Golden eagles on the Swedish mountain tundra - Diet and breeding success in relation to prey fluctuations. *Ornis Fennica*, *83*(4), 145–152.

Obuch, J., & Bangjord, G. (2016). The Eurasian eagle-owl (Bubo bubo) diet in the Trøndelag region (Central Norway). *Slovak Raptor Journal*, *10*(1), 51–64. https://doi.org/10.1515/srj-2016-0005

Obuch, J., & Karaska, D. (2010). The Eurasian eagle-owl (Bubo bubo) diet in the Orava Region (N Slovakia). *Slovak Raptor Journal*, *4*(1), 83–98. https://doi.org/10.2478/v10262-012-0048-9

Odden, J., Linnell, J. D. C., & Andersen, R. (2006). Diet of Eurasian lynx, Lynx lynx, in the boreal forest of southeastern Norway: the relative importance of livestock and hares at low roe deer density. *European Journal of Wildlife Research*, *52*(4), 237–244. https://doi.org/10.1007/s10344-006-0052-4

Okarma, H., Jȩdrzejewski, W., Schmidt, K., Kowalczyk, R., & Jȩdrzejewska, B. (1997). Predation of Eurasian lynx on roe deer and red deer in Białowieża Primeval Forest. *Acta Theriologica*, *42*(2), 203–224. https://doi.org/10.4098/AT.arch.97-22

Olech, B. (1997). Diet of the Goshawk Accipiter gentilis in Kampinoski National Park (Central Poland) in 1982-1993. *Acta Ornithologica*, *32*, 191–200.

Opdam, P., Thissen, J., Verschuren, P., & Müskens, G. (1977). Feeding ecology of a population of Goshawk Accipiter gentilis. *Journal of Ornithology*, *118*(1), 35–51. https://doi.org/10.1007/BF01647356

Pagh, S., Tjørnløv, R. S., Olesen, C. R., & Chriel, M. (2015). The diet of Danish red foxes (Vulpes vulpes) in relation to a changing agricultural ecosystem. A historical perspective. *Mammal Research*, *60*(4), 319–329. https://doi.org/10.1007/s13364-015-0244-y

Panzacchi, M., Linnell, J. D. C., Serrao, G., Eie, S., Odden, M., Odden, J., & Andersen, R. (2008). Evaluation of the importance of roe deer fawns in the spring–summer diet of red foxes in southeastern Norway. *Ecological Research*, *23*(5), 889–896. https://doi.org/10.1007/s11284-007-0452-2

Pasanen‐Mortensen, M., Elmhagen, B., Lindén, H., Bergström, R., Wallgren, M., Velde, Y., & Cousins, S. A. O. (2017). The changing contribution of top‐down and bottom‐up limitation of mesopredators during 220 years of land use and climate change. *Journal of Animal Ecology*, *86*(3), 566–576. https://doi.org/10.1111/1365-2656.12633

Pasanen-Mortensen, M., Pyykönen, M., & Elmhagen, B. (2013). Where lynx prevail, foxes will fail - limitation of a mesopredator in Eurasia. *Global Ecology and Biogeography*, *22*(7), 868–877. https://doi.org/10.1111/geb.12051

Pedrini, P., & Sergio, F. (2001). Density, productivity, diet, and human persecution of golden eagles (Aquila chrysaetos) in the central-eastern Italian Alps. *Journal of Raptor Research*, *35*(1), 40–48.

Petty, S. J., Anderson, D. I. K., Davison, M., Little, B., Sherratt, T. N., Thomas, C. J., & Lambin, X. (2003). The decline of Common Kestrels Falco tinnunculus in a forested area of northern England: the role of predation by Northern Goshawks Accipiter gentilis. *Ibis*, *145*(3), 472–483. https://doi.org/10.1046/j.1474-919X.2003.00191.x

Pinn, G. (1967). Die nahrung und jagd des Steinadlers [In German]. *Anzeiger Der Ornithologische Gesellschaft in Bayern*, *8*, 59–62.

Posłuszny, M., Pilot, M., Goszczyński, J., & Gralak, B. (2007). Diet of sympatric pine marten (Martes martes) and stone marten (Martes foina) identified by genotyping of DNA from faeces. In *Annales Zoologici Fennici* (Vol. 44, Issue 4).

Pulliainen, E. (1981). Winter diet of Felis lynx in SE Finland as compared with the nutrition of other northern lynxes. *Zeitschrift Fuer Saeugetierkunde*, *46*(4), 249–259. Pulliainen_1981_Winter_diet_of_lynx_in_Finland.pdf

Pulliainen, E., Lindgren, E., & Tunkkari, P. S. (1995). Influence of food availability and reproductive status on the diet and body condition of the European lynx in Finland. *Acta Theriologica*, *40*(2), 181–196. https://doi.org/10.4098/AT.arch.95-19

Pulliainen, E., & Ollinmäki, P. (1996). A long-term study of the winter food niche of the pine marten Martes martes in northern boreal Finland. *Acta Theriologica*, *41*(4), 337–352. https://doi.org/10.4098/AT.arch.96-33

Reif, V., Tornberg, R., Jungell, S., & Korpimäki, E. (2001). Diet variation of common buzzards in Finland supports the alternative prey hypothesis. *Ecography*, *24*(3), 267–274. https://doi.org/10.1034/j.1600-0587.2001.240304.x

Rooney, E., & Montgomery, W. I. (2013). Diet diversity of the Common Buzzard (Buteo buteo) in a vole-less environment. *Bird Study*, *60*(2), 147–155. https://doi.org/10.1080/00063657.2013.772085

Russell, A. J. M., & Storch, I. (2004). Summer food of sympatric red fox and pine marten in the German Alps. *European Journal of Wildlife Research*, *50*(2), 53–58. https://doi.org/10.1007/s10344-004-0037-0

Sándor, A. D., & Ionescu, D. T. (2009). Diet of the eagle owl (Bubo bubo) in Braşov, Romania. *North-Western Journal of Zoology*, *5*(1), 170–178.

Sándor, D. A., & Bugariu, S. (2008). Food habits of the Eurasian Eagle Owl (Bubo bubo) in Cheile Dobrogei Gorge. *Scientific Annals of the Danube Delta Institute*, *14*, 69–74.

Schaefer, H. (1971). Beutetiere des Uhus Buho buho aus Karpaten und Lappland [In German]. *Bonner Zoologische Beiträge*, *22*, 153–160.

Selås, V. (2001). Predation on reptiles and birds by the common buzzard, Buteo buteo , in relation to changes in its main prey, voles. *Canadian Journal of Zoology*, *79*(11), 2086–2093. https://doi.org/10.1139/z01-183

Selås, V., Tveiten, R., & Aanonsen, O. M. (2007). Diet of Common Buzzards (Buteo buteo) in southern Norway determined from prey remains and video recordings. *Ornis Fennica*, *84*(3), 97–104.

Sergio, F., Boto, A., Scandolara, C., & Bogliani, G. (2002). Density, nest sites, diet, and productivity of Common Buzzards (Buteo buteo) in the Italian pre-Alps. *Journal of Raptor Research*, *36*(1), 24–32.

Sergio, F., Marchesi, L., Pedrini, P., & Penteriani, V. (2007). Coexistence of a generalist owl with its intraguild predator: distance-sensitive or habitat-mediated avoidance? *Animal Behaviour*, *74*(6), 1607–1616. https://doi.org/10.1016/j.anbehav.2006.10.022

Ševčík, J. (1980). Potrava káně lesní (Buteo buteo), káně rousné (Buteo lagopus) a jestřába lesního (Accipiter gentilis) v oblastech s intenzivním chovem bažantů [In Czech]. *Sylvia*, *20*, 35–43.

Sidorovich, A. A., Ivanovskij, V. V., Sidorovich, V. E., & Solovej, I. A. (2016). Landscape-related variation in the diet composition of the common buzzard (Buteo buteo) in Belarus. *Slovak Raptor Journal*, *10*(1), 65–74. https://doi.org/10.1515/srj-2016-0006

Sidorovich, V. E., Sidorovich, A. A., & Izotova, I. V. (2006). Variations in the diet and population density of the red fox Vulpes vulpes in the mixed woodlands of northern Belarus. *Mammalian Biology*, *71*(2), 74–89. https://doi.org/10.1016/j.mambio.2005.12.001

Sidorovich, V. (2011). Predation on predators. In *Analysis of vertebrate predator-prey community* (pp. 262–277). Tesey. https://www.researchgate.net/publication/261925440_Analysis_of_vertebrate_predator-prey_community_Studies_within_the_European_Forest_zone_in_terrains_with_transitional_mixed_forest_in_Belarus

Sim, I. M. W., Cross, A. V., Lamacraft, D. L., & Pain, D. J. (2001). Correlates of common buzzard buteo buteo density and breeding success in the west midlands. *Bird Study*, *48*(3), 317–329. https://doi.org/10.1080/00063650109461231

Skouen, S. K. (2012). *Assessing diet and prey handling in golden eagles Aquila chrysaetos by video monitoring at nest* [Norwegian University of Life Sciences]. http://hdl.handle.net/11250/187123

Sládek, J. (1961). Príspevok k poznaniu potravnej ekológie myšiaka lesného Buteo buteo (L.) [In Slovak]. *Zool Listy*, *10*(1), 331–343.

Smedshaug, C. A., Selås, V., Lund, S. E., & Sonerud, G. A. (1999). The effect of a natural reduction of red fox Vulpes vulpes on small game hunting bags in Norway. *Wildlife Biology*, *5*(3), 157–166. https://doi.org/10.2981/wlb.1999.020

Sonerud, G. A. (1985). Risk of nest predation in three species of hole nesting owls: influence on choice of nesting habitat and incubation behaviour. *Ornis Scandinavica*, *16*(4), 261–269. https://doi.org/10.2307/3676689

Šotnár, K., & Obuch, J. (2009). Feeding ecology of a nesting population of the Common Buzzard (Buteo buteo) in the Upper Nitra region, Central Slovakia. *Slovak Raptor Journal*, *3*(1), 13–20. https://doi.org/10.2478/v10262-012-0028-0

Storch, I., Lindström, E., & Jounge, J. de. (1990). Diet and habitat selection of the pine marten in relation to competition with the red fox. *Acta Theriologica*, *35*, 311–320. https://doi.org/10.4098/AT.ARCH.90-36

Sulkava, S., Huhtala, K., Rajala, P., & Tornberg, R. (1999). Changes in the diet of the Golden Eagle Aquila chrysaetos and small game populations in Finland in 1957-96. *Ornis Fennica*, *76*(1), 1–16.

Sunde, P. (2005). Predators control post-fledging mortality in tawny owls, Strix aluco. *Oikos*, *110*(3), 461–472. https://doi.org/10.1111/j.0030-1299.2005.14069.x

Sunde, P., & Kvam, T. (1997). Diet patterns of Eurasian lynx Lynx lynx: What causes sexually determined prey size segregation? *Acta Theriologica*, *42*(2), 189–201. https://doi.org/10.4098/AT.arch.97-21

Sunde, P., Kvam, T., Bolstad, J. P., & Bronndal, M. (2000). Foraging of Lynxes in a Managed Boreal-Alpine Environment. *Ecography*, *23*(3), 291–298. http://www.jstor.org/stable/3683099

Sunde, P., Overskaug, K., & Kvam, T. (1999). Intraguild predation of lynxes on foxes: evidence of interference competition? *Ecography*, *22*(5), 521–523. https://doi.org/10.1111/j.1600-0587.1999.tb01281.x

Swann, R. L., & Etheridge, B. (1995). A comparison of breeding success and prey of the common buzzard buteo buteo in two areas of northern scotland. *Bird Study*, *42*(1), 37–43. https://doi.org/10.1080/00063659509477146

Tjernberg, M. (1981). Diet of the golden eagle Aquila chrysaetos during the breeding season in Sweden. *Ecography*, *4*(1), 12–19. https://doi.org/10.1111/j.1600-0587.1981.tb00975.x

Tornberg, R., Mönkkönen, M., & Kivelä, S. M. (2009). Landscape and season effects on the diet of the Goshawk. *Ibis*, *151*(2), 396–400. https://doi.org/10.1111/j.1474-919X.2009.00910.x

Toyne, E. P. (1998). Breeding season diet of the Goshawk Accipiter gentilis in Wales. *Ibis*, *140*(4), 569–579. https://doi.org/10.1111/j.1474-919X.1998.tb04701.x

Twining, J. P., Montgomery, I., Fitzpatrick, V., Marks, N., Scantlebury, D. M., & Tosh, D. G. (2019). Seasonal, geographical, and habitat effects on the diet of a recovering predator population: the European pine marten (Martes martes) in Ireland. *European Journal of Wildlife Research*, *65*(3), 1–15. https://doi.org/10.1007/s10344-019-1289-z

Uttendörfer, O. (1952). *Neue Ergebnisse über die Ernährung der Greifvögel und Eulen [In German]*. Verlag J Neumann, Neudamm.

Uttendörfer, O. (1939). *Die Ernährung der deutschen Raubvögel und Eulen und ihre Bedeutung in der heimischen Natur [In German]*. Verlag J Neumann, Neudamm.

Valdmann, H., Andersone-Lilley, Z., Koppa, O., Ozolins, J., & Bagrade, G. (2005). Winter diets of wolf Canis lupus and lynx Lynx lynx in Estonia and Latvia. *Acta Theriologica*, *50*(4), 521–527. https://doi.org/10.1007/BF03192645

Village, A. (1981). The diet and breeding of long-eared owls in relation to vole numbers. *Bird Study*, *28*(3), 215–225. https://doi.org/10.1080/00063658109476726

Voříšek, P., Krištín, A., Obuch, J., & Votypka, J. (1997). Diet of Common Buzzard in the Czech Republic and its importance for game keeping. *Buteo*, *9*, 57–68.

Wassink, G. (2003). Eerste broedgeval van Oehoe Bubo bubo in de Achterhoek. *Limosa*, *76*, 1–10.

Watson, J., Leitch, A. F., & Broad, R. A. (1992). The diet of the Sea Eagle Haliaeetus albicilla and Golden Eagle Aquila chrysaetos in western Scotland. *Ibis*, *134*(1), 27–31. https://doi.org/10.1111/j.1474-919X.1992.tb07225.x

Watson, J. (1998). Should Golden Eagles Aquila chrysaetos be Food Generalists or Specialists? In B. U. Meyburg, R. D. Chancellor, & J. J. Ferrero (Eds.), *Holartic Birds of Prey*. World Working Group on Birds of Prey and Owls. http://www.raptors-international.org/book/holarctic_birds_of_prey_1998/Watson_1998_251-261.pdf

Webbon, C. C., Baker, P. J., Cole, N. C., & Harris, S. (2006). Macroscopic prey remains in the winter diet of foxes Vulpes vulpes in rural Britain. *Mammal Review*, *36*(1), 85–97. https://doi.org/10.1111/j.1365-2907.2006.00069.x

Widén, P. (1987). Goshawk predation during winter, spring and summer in a boreal forest area of central Sweden. *Ecography*, *10*(2), 104–109. https://doi.org/10.1111/j.1600-0587.1987.tb00745.x

Wikenros, C., Aronsson, M., Liberg, O., Jarnemo, A., Hansson, J., Wallgren, M., Sand, H., & Bergström, R. (2017). Fear or food – abundance of red fox in relation to occurrence of lynx and wolf. *Scientific Reports*, *7*(1), 9059. https://doi.org/10.1038/s41598-017-08927-6

Wikenros, C., Ståhlberg, S., & Sand, H. (2014). Feeding under high risk of intraguild predation: vigilance patterns of two medium-sized generalist predators. *Journal of Mammalogy*, *95*(4), 862–870. https://doi.org/10.1644/13-MAMM-A-125

Wilke, M., Gottman, A., & Schneider, H. G. (1985). Beutetiere des Habichts (Accipiter gentilis) auf drei nordhessischen Untersuchungsflächen. *Vogelkundliche*, *11*, 69–77.

Wuttky, K. (1963). Beutetier-Funde in Greifvögelhorsten des Hakel [In German]. *Beitr Vogelkd*, *93*, 140–171.

Zárybnická, M., Sedláček, O., Salo, P., Šťastný, K., & Korpimäki, E. (2015). Reproductive responses of temperate and boreal Tengmalm’s Owl Aegolius funereus populations to spatial and temporal variation in prey availability. *Ibis*, *157*(2), 369–383. https://doi.org/10.1111/ibi.12244

Zastrov, M. (1946). Om kungsörnens Aguila chrysaetos ut bredning och biologi i Estland [In Swedish]. *Vâr Fagelvärld*, *5*, 64–80.

Zawadzka, D., & Zawadzki, J. (1998). The goshawk Accipiter genitlis in Wigry National Park (NE Poland) - numbers, breeding results, diet composition and prey selection. *Acta Ornithologica*, *33*, 181–190.

Zoltan, P., & Bela, S. (2014). Adatok az uhu (Bubo bubo) északkelet-magyarországi állományának táplálkozásához [In Hungarian]. *Heliaca*, 98–103.

**TABLE S5. Complete list of credits for silhouettes used in figure 1. All were obtained from** [**http://phylopic.org/**](http://phylopic.org/)**.**

| Species | Copyright | Credit | Link to License | Copy | | Distribute | | Transmit | | Adapt | | Non-commercial | | Share Alike |  |
| --- | --- | --- | --- | --- | --- | --- | --- | --- | --- | --- | --- | --- | --- | --- | --- |
| pine marten | y | Anthony Caravaggi | <https://creativecommons.org/licenses/by-nc-sa/3.0/> | y | | y | | y | | y | | y | | y |  |
| golden eagle | y | Anthony Caravaggi | <https://creativecommons.org/licenses/by-nc-sa/3.0/> | y | | y | | y | | y | | y | | y |  |
| raven | y | Anthony Caravaggi | <https://creativecommons.org/licenses/by-nc-sa/3.0/> | y | | y | | y | | y | | y | | y |  |
| kestrel | y | Liftarn | <https://creativecommons.org/licenses/by-nc-sa/3.0/> | y | | y | | y | | y | | y | | y |  |
| Eagle owl | y | Lukasiniho | <https://creativecommons.org/licenses/by-nc-sa/3.0/> | y | | y | | y | | y | | y | | y |  |
| red fox | n |  |  |  | |  | |  | |  | |  | |  |  |
| common buzzard | n |  |  |  | |  | |  | |  | |  | |  |  |
| accipiter | n |  |  | |  | |  | |  | |  | |  | |  |
| carrion crow | n |  |  | |  | |  | |  | |  | |  | |  |
| kestrel | n |  |  | |  | |  | |  | |  | |  | |  |
| Tawny owl | n |  |  | |  | |  | |  | |  | |  | |  |
| least weasel | n |  |  | |  | |  | |  | |  | |  | |  |
| stoat | n |  |  | |  | |  | |  | |  | |  | |  |
| lynx | n |  |  | |  | |  | |  | |  | |  | |  |
| grouse | n |  |  | |  | |  | |  | |  | |  | |  |

**FIGURE** **S6**. **Distribution of data: 107 locations from 160 studies. White triangles: diet studies; Empty circles: studies with killings or mortality rates; Black squares: studies with evidence of population suppression (or lack thereof). Where the exact location of a study was not available it was attributed to central coordinates within its region or country. Grey filled symbols represent studies with large geographical remits (from east to west: British Isles, central and western Europe, Fennoscandia, Eurasia).**


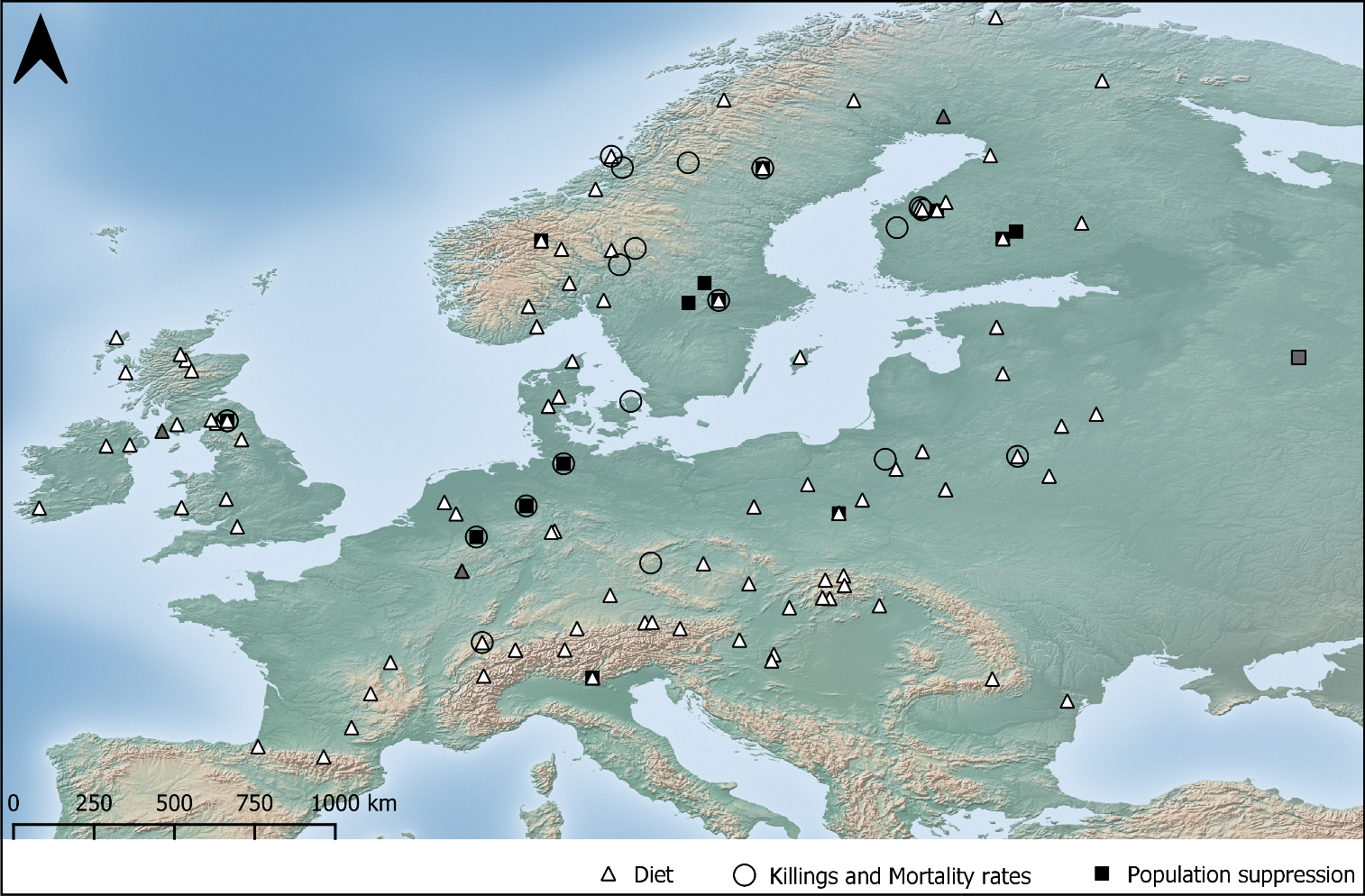

Supplement: Supplementary file 4 — Appendix S1 [file ECE3-11-7164-s004.docx]
